# Transcriptomic Insights into Micro- and Nanoplastic Toxicity in Zebrafish: A Narrative Review

**DOI:** 10.3390/toxics14070572

**Published:** 2026-06-29

**Authors:** Nikita A. Mitkin, Aleksey A. Vatlin, Svetlana N. Nikulina, Elohor O. Amarie, Vsevolod V. Pavshintsev

**Affiliations:** Institute of Environmental Engineering of RUDN University, 6 Miklukho-Maklaya St, Moscow 117198, Russia

**Keywords:** microplastics, nanoplastics, zebrafish, transcriptome

## Abstract

Micro- and nanoplastics (MNPs) are emerging global pollutants that pose a significant threat to living organisms due to their widespread presence, ingestion by aquatic species, and ability to cross biological barriers, including the blood–brain barrier. Zebrafish is a well-established and convenient model for ecotoxicological research because of its small size, optical transparency, fully sequenced genome, high genetic homology to humans, ease of breeding, and short life cycle. Exposure to MNPs affects multiple organ systems in zebrafish, including the brain, eyes, liver, intestine, gills, and reproductive system. These particles can induce oxidative stress, inflammation, and interference with diverse biomolecules, leading to adverse biological effects. An analysis of transcriptomic alterations induced by MNPs exposure can contribute to understanding the mechanisms of these adverse effects. In this narrative review, we classify existing studies on MNPs exposure in zebrafish by affected organ system and summarize the gene expression-based evidence of MNP-induced toxicity with a particular focus on high-throughput approaches such as RNA sequencing and single-cell RNA sequencing.

## 1. Introduction

Plastic materials are widely used in our daily lives due to their versatility, light weight, and low cost. These properties have led to their widespread application across virtually all sectors from packaging and construction to microplastic beads in personal care products [1,2]. Consequently, global plastic production has increased dramatically over the last 6 years from 370 to 430 million tons [3]. A significant portion of this plastic waste is improperly managed, leading to its accumulation in terrestrial, aquatic, and even atmospheric environments [4,5]. Over time, larger plastic debris fragments into progressively smaller particles through various physical, chemical, and biological degradation processes, giving rise to what are now recognized as microplastics (MPs) and nanoplastics (NPs). MPs are defined as particles ≤5 mm in size and are now recognized as a highly diverse set of globally important contaminants. Plastic particles smaller than 1 µm are regarded as NPs [6]. Micro- and nanoplastics (MNPs) are omnipresent; the presence of MPs has been observed not only in soil, fresh and marine water environments but also in the Mount Everest summit snow [7], Arctic marine sediments [8], in the Mariana Trench [9] and in the human body [10]. MPs originate from two main categories: primary and secondary sources. Primary MPs are intentionally produced for industrial (road markings, marine coatings) [11], agricultural (fertilizers) [12], and commercial applications (personal care products, air blasting) [13]. Secondary MPs originate from the breakdown of larger plastic items, distinguishing them from primary MPs, which are manufactured at smaller sizes. The degradation of plastic occurs through abrasion, UV radiation (photodegradation), heat, mechanical action, or biological processes such as biofilm formation by bacteria [14,15]. Polypropylene (PP), polyethene terephthalate (PET), low- and high-density polyethene (PE), polystyrene (PS), polyurethane, and polyvinyl chloride are the primary plastic polymers produced by the manufacturing sector today [16].

A meta-analysis of 199 studies reporting MP (100 μm threshold) abundance in water bodies found that abundance was highest in Africa followed by Asia, Europe, the Americas, and Oceania [17]. The local level of MP contamination depends on industrial activities and wastewater discharge. MP has been identified at varying concentrations worldwide, for example, in the Flemish river (Belgium) at 0.0153 g/L [18], 0.26685 g/L in the daily flow of a tertiary wastewater treatment plant (UK) [19], 1.7397 g/L in Winnipeg lake (Canada), 2.106 g/L in sediments in the lagoon of Bizerte (Tunisia), 2.1–71.0 particles/L in industrial wastewater near a textile zone (China) [20], and 68–568 particles/L in runoff from industrial areas (Gumi, South Korea) [21]. In freshwater systems, MNPs have been consistently detected in economically and ecologically important fish species, including common carp (*Cyprinus carpio*) [22], rainbow trout (*Oncorhynchus mykiss*) [23], and gudgeons (*Gobio gobio*) [24]. MNPs can affect the health, survival, and reproductive success of freshwater fish and other vertebrates, disrupt the food chain, lead to MNP accumulation through the food chain, and worsen the economy [25]. MNPs have been found in fish gills, gut, liver, heart, muscles, swim bladders, ovaries, spinal cords, eyes, and even brains [26]. The detection of MNPs in these organs has been associated with marked harmful outcomes, such as tissue damage, impaired reproduction, developmental impairments, neurotoxicity, hormonal imbalance, dysregulation of immune function [26,27] and gut dysbiosis [28]. Moreover, recent studies have identified the presence of MNPs in different parts of the human body, such as the placenta [10], lung tissue [29], brain [30] and blood [31]. Interestingly, MP abundance was even greater in brain samples from dementia cases, which might point to a possible link between MPs and dementia [30].

The exposure of living organisms to MNPs poses an emerging threat to biological systems across multiple trophic levels, necessitating a comprehensive investigation of the mechanisms by which these particles exert toxic effects. Understanding MNPs’ toxicity at the molecular level is particularly valuable for elucidating how these contaminants disrupt fundamental biological processes and compromise organismal health. To advance this understanding, this review synthesizes existing transcriptomic studies to systematically evaluate how exposure to distinct MNPs, including polymer types, sizes, and formulations, influences gene expression patterns in zebrafish (*Danio rerio*), which is a widely utilized model organism for developmental and toxicological investigations.

## 2. Materials and Methods

A PubMed search was performed using combinations of terms for zebrafish (zebrafish, *Danio rerio*), transcriptomic outcomes (transcriptome, transcriptomic, RNA-seq, gene expression), and plastic exposures (microplastics, nanoplastics, and common polymer types such as polystyrene, polyethylene, polypropylene, PVC, PET). Searches were restricted to title and abstract fields to improve relevance. The search was performed using the Entrez Programming Utilities (E-utilities) API through Python (v. 3.9.13) with the Biopython library. Initial database searches identified 160 relevant articles. After screening, studies involving co-exposure experiments and non-relevant articles were excluded. This filtering process resulted in 60 articles that were considered relevant for the review.

## 3. Zebrafish as a Model for the Investigation of MNP Effects with Transcriptomics

Zebrafish embryos and larvae are optically transparent, a major advantage for studying MPs and NPs, as fluorescently labeled particles can be tracked in real time within the organism. The accumulation, distribution, and translocation of MNP across biological barriers can be directly visualized without dissection. With the application of fluorescent-labeled MNPs, accumulation was reported in the yolk sac [32] and chorion [33] of zebrafish embryos, as well as in the eye tissue [34], brain [35], liver [36], gills [37], gastrointestinal tract [38], heart [39] and at the injury site [32] in the larvae and adult zebrafish. Additionally, because embryo and larval organ development is transparent, damage can be monitored non-invasively under a microscope [40,41].

Zebrafish have rapid development and high fecundity. Female zebrafish lay approximately 100–200 eggs per spawning, providing a large sample size for statistical analysis [42]. The development of a fully functional larva takes 72–120 h post-fertilization (hpf), and development is complete around 45 days post-fertilization (dpf) when fish are grown under optimal conditions [43]. That allows access to developmental toxicity through all life stages. Additionally, generation times are about 3 months, enabling multigenerational studies to assess how maternal exposure to MP/NP affects offspring [44].

Zebrafish naturally inhabit freshwater environments, which are most heavily contaminated with MNPs from runoff, wastewater, and the degradation of plastic debris [45]. Therefore, as a freshwater vertebrate, zebrafish may respond to pollutants similarly to other aquatic organisms [46].

Zebrafish offer a wide variety of measurable endpoints relevant to MPs and NPs toxicity. For example, the impact of MNPs on embryonic development can be assessed using hatching rate, malformations, survival, and heart rate. Moreover, the neurotoxicity of MNPs in larval and adult zebrafish can be evaluated by studying locomotor behavior (swimming activity), and certain patterns of altered locomotor activity can be translated into attention-deficit/hyperactivity disorder (ADHD) [47].

The zebrafish genome is fully sequenced and exhibits 70% homology with the human genome [48], enabling the precise identification of genes and pathways affected by MNPs and the translation of these changes to mammals. Genetic manipulation (knockouts, transgenics, CRISPR-Cas9) in zebrafish allows researchers to identify specific molecular targets of MNPs [49]. Zebrafish has been widely used as a non-mammalian model to study infectious diseases, particularly sensing pathways involved in the innate immune response [50].

Transcriptomic analysis is now widely used to assess the toxicity caused by MNPs. Capturing genome-wide changes in mRNA abundance after exposure to xenobiotic stressors enables a detailed characterization of cellular stress programs and reveals which signaling pathways and regulatory networks are disrupted [51].

Transcriptomic methods frequently applied in toxicology studies include targeted gene expression analysis, such as quantitative reverse transcription PCR (qRT-PCR) and microarrays, as well as untargeted approaches, such as RNA sequencing (RNA-seq) and single-cell RNA sequencing (scRNA-seq). qRT-PCR is widely used for the targeted quantification of specific biomarker genes related to oxidative stress, inflammation, TGF-beta signaling, and other processes [52,53]. Microarrays are based on the hybridization of fluorescently labeled cDNA to pre-designed oligonucleotide or cDNA probes immobilized on a chip [54]. Microarrays measure the expression of pre-annotated gene sets, which limits analysis to several pathways. By contrast, RNA-seq offers an unbiased, genome-wide quantification of transcripts. Currently, it is the most widely used transcriptomic approach in ecotoxicology to assess changes in global gene expression levels. Additionally, it allows the discovery of novel transcripts, splice variants, and non-coding RNAs. scRNA-seq is a more advanced analysis than bulk RNA-seq. scRNA-seq profiles transcriptomes at single-cell resolution to detect cell-type-specific toxicant responses and shifts in cell populations [55,56]. In the majority of studies included in this review, RNA-seq is the most widely used method to investigate alterations in gene expression. One of the most critical challenges in MNP research is understanding the underlying mechanisms of toxicity rather than merely describing phenotypic outcomes.

Applying transcriptomics in MNPs studies is valuable because it can reveal early, system-wide biological changes and help to identify the mechanisms through which MNPs cause toxicity and phenotypic changes. Transcriptomics enables the identification of dysregulated genes, biological pathways, and molecular networks, such as oxidative stress, inflammatory responses, endocrine disruption, neurotoxicity, DNA damage, apoptosis and metabolic disruption [38,53,57].

### 3.1. Hematopoietic System

Previous research has demonstrated that exposure to NPs impairs hematopoietic cell function in the mouse bone marrow, reducing self-renewal and differentiation capacity [58,59].

NPs caused caudal vein plexus (CVP) damage after 46 h of exposure at concentrations of 5 and 8 mg/L [60]. The CVP provides a supportive microenvironment for the maturation, proliferation, and differentiation of hematopoietic stem/progenitor cells (HSPCs) [61]. RNA-seq identified transcriptional changes associated with the upregulation of positive regulation of nuclear cell cycle DNA replication, epithelial–mesenchymal cell signaling, and the positive regulation of neutrophil activation in the group exposed to 5 mg/L of NPs. In the group exposed to 8 mg/L of NPs, pathways such as centriole elongation, nucleotide excision repair DNA gap filling, and negative regulation of glycogen metabolism were upregulated. An enhanced positive regulation of neutrophil activation indicates immune system activation, while alterations in pathways such as centriole elongation and nucleotide excision repair DNA gap filling affect cell division and DNA damage repair capacity. scRNA-seq analysis of zebrafish embryo tails identified 22 distinct cell clusters with HSPCs and epithelial cells (ECs) representing the most abundant subpopulations. Notably, enrichment analysis revealed a relationship between EC mitosis and cellular redox status: concurrent enrichment in mitotic processes (mitotic cell cycle progression, organelle fission, mitotic nuclear division) and redox-regulatory activities (tubulin binding, antioxidant activity, oxidoreductase activity). HSPC enrichment analysis showed an activation of protein synthesis, enhanced antioxidant activity, and peroxidase activity, highlighting the critical role of redox regulation in HSPC homeostasis [60].

The head kidney is the primary site of hematopoiesis and one of the most prominent lymphoid organs in teleost fish. Alongside the spleen, it plays a crucial role in filtering the blood [62]. MPs have been found to alter the expression of c*yp*2*p*8 (xenobiotic catabolism) and of *tcra*, which are both related to immune response [63].

Exposure to PS-NPs (20 nm) reduced the abundance of RBCs in zebrafish embryos. Pathway enrichment analysis of the RBC cluster revealed a global suppression of translation- and differentiation-related pathways as well as heme synthesis. Among translation-associated genes, rps7 showed the most pronounced decrease, and its knockdown further confirmed the reduction in RBCs. Other findings were observed in RBC sub-clusters: in the PS-NP-exposed group, the proportion of common myeloid progenitors (CMPs) was higher, which was accompanied by a significant reduction in the proportion of mature erythrocytes compared with the control [49]. CMP is the stage of differentiation from hematopoietic stem cells to erythrocytes [64]. A reduced expression of genes associated with heme synthesis implies a reduced availability of heme metabolites, which are required for erythropoiesis. Together, NP exposure inhibited heme synthesis and translation, leading to disrupted differentiation and the accumulation of immature erythrocytes [49]. Although the toxic effects of PS-NPs are clearly observed, the molecular mechanism underlying altered gene expression needs further elucidation.

**Figure 1:**
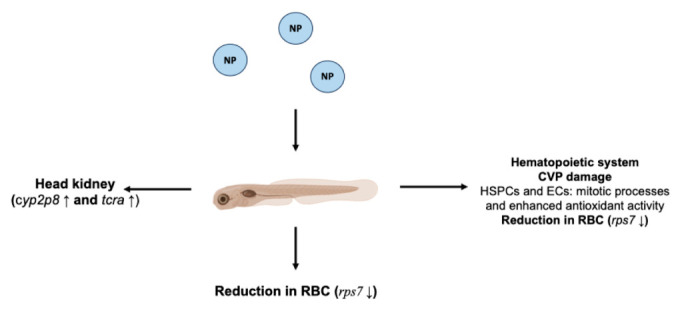
NPs exposure impacts on hematopoietic system and head kidney in zebrafish larvae.

Together, these findings demonstrated the toxic effects of NPs on erythropoiesis in zebrafish embryos (Figure 1).

### 3.2. Cardiovascular System

PE and PS-MPs have been found to cause cardiotoxicity in adult and embryonic zebrafish [65,66] as well as in mice and human-derived cardiac organoid models [67].

It has been shown by Saputra et al. (2025) that PS-NP induced cardiovascular impairments in larval zebrafish. Even 0.1 μg/mL of 25 nm PS-NP declined the expression of genes crucial for cardiovascular development, such as *gata* family members, *nkx*2.5/2.7, *hand*2, *tbx*2*a/b*, and *fgf*1*a.* Additionally, PS-NP induced the expression of pro-apoptotic genes (*tp53*, *casp*3, *casp*9, *bax*) and oxidative stress-related genes, which was accompanied by an increased production of ROS and induced apoptosis. Possibly, PS-NPs induce oxidative stress, subsequently causing cell apoptosis [68].

In the study by Liu et al. (2024), 4- and 7-day exposure of zebrafish embryos to 80 nm PS-NPs resulted in heart malformations, including pericardial edema, while concentrations of 10 μg/mL and 100 μg/mL significantly increased the incidence of cardiac abnormalities in zebrafish larvae. qRT-PCR showed that PS-NP exposure induced broad transcriptional changes in zebrafish larvae, indicating disrupted cardiac development and cellular homeostasis. Heart development-related genes *nkx*2.5, *cmlc-*2, and *myh-*7 were upregulated, whereas *gata-*4 was downregulated. *Gata-*4, a transcription factor, is involved in regulating cardiomyocyte differentiation during heart development; altered *Gata*-4 regulation suggests impaired cardiogenesis [69]. *Nkx*2.5 plays a central role in determining myocardial cell fate [70]. The initiation of *cmlc-2* expression drives the differentiation of cardiomyocytes into the atrial and ventricular subgroups [71]. In parallel, PS-NPs activated the Notch pathway by increasing the expression of *notch-*1*a*, *jag-*1*a*, and *her-*7 while partially suppressing Wnt signaling through reduced *wnt-*3*a* expression. Wnt and Notch are both required for normal heart development [72,73]. Moreover, in zebrafish, a reduction in mitochondrial copy number and a significant decrease in Ca^2+^ concentration were observed, indicating altered functions of mitochondria and the endoplasmic reticulum, which is the main reservoir of Ca^2+^. At the mitochondrial level, the reduced expression of *mt-nd1* and *mt-cyb,* together with lower levels of superoxide dismutase*,* suggests weakened function and antioxidant defense. The upregulation of *atf-*6, *chop*, and *xbp-*1 points to endoplasmic reticulum stress, which was supported by a sustained decrease in Ca^2+^ levels. Overall, the authors proposed a mechanistic explanation of PS-NP toxicity in which PS-NP-induced oxidative stress triggers endoplasmic reticulum stress and reduces the mitochondrial copy number. These disturbances likely contribute to dysregulation of the Notch and Wnt signaling pathways, ultimately leading to abnormal cardiac development in zebrafish larvae [74].

Kim et al. (2025) investigated whether the chorion provides a protective barrier against MPs by exposing zebrafish embryos to PE-MPs larger than the chorion’s pores. The authors observed that even without dechorionation, 1–4 µm PE-MPs can affect embryo development. The heart rate was significantly reduced in a dose-dependent manner across all PE-treated groups compared with the control, indicating the cardiotoxicity of PE-MPs in zebrafish embryos. After 42 h of exposure to an environmentally relevant concentration (1 mg/L), the authors performed RNA-seq on the embryos, and subsequent GO analysis revealed a significant downregulation of genes associated with heart development. RT-qPCR confirmed that *fbln*1 and *fn*1*b* were significantly reduced in the PE-treated group compared with the control group. Protein–protein interaction analysis to investigate the role of *fbln* in heart development identified proteins Itga3a, Itga3b, and Vtnb, which are associated with heart development. Reduced expression levels of *itga*3*a*, *itga*3*b*, and *vtnb* further confirmed a significant downregulation of heart development-related genes in the PE-treated group compared with the control. The authors suggest that the decrease in *fn*1*b* expression indicates that PE-MP exposure may interfere with extracellular matrix formation and cell adhesion processes essential for proper heart morphogenesis [75].

Polylactic acid (PLA) is a bio-based and biodegradable plastic considered less harmful to the environment. However, PLA-MPs have been found to accumulate in aquatic ecosystems and affect living organisms. For example, they have caused oxidative stress, inflammation, and alterations in the hearts of zebrafish larvae [76,77,78]. PLA-MP exposure activated PPARγ, which upregulated key metabolic genes—*adipoq*, *cyp*11*a*1, *fabp*11*a*, *lpl*, *pnpla*2, *cd*36, and *lipea*—driving enhanced lipid metabolism in zebrafish larvae. This resulted in impaired cardiac morphology and function, suppressed liver development, and a dysregulation of core cardiac developmental genes, including *myl*7, *gata*4, *sox*9*b*, and *tbx*5*a* [78].

**Figure 2:**
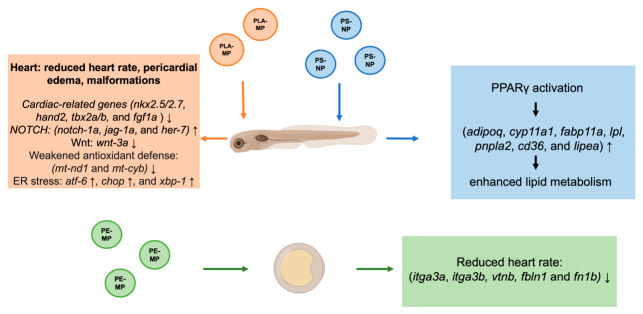
MNPs exposure impacts om cardiovascular system of zebrafish.

A summary of the altered genes and pathways caused by MNPs is presented in Figure 2.

### 3.3. Neurotoxicity

PS-MNPs induced neurodevelopmental impairments in larval zebrafish via oxidative stress and disruption of the neurotransmitter system [79].

LeMoine et al. (2018) studied the influence of PE-MPs on the early life stages of zebrafish. No toxic effects were observed in hatching, mortality, growth, or O_2_ consumption. Zebrafish larvae were exposed to PE-MPs for 48 h and 14 days at two concentrations. Gene expression analysis of whole organisms revealed that PE-MP exposure induced substantial transcriptional changes in zebrafish larvae, particularly during acute exposure. RNA-seq showed that 48 h of MP exposure (5 and 20 mg/L) resulted in 1734 and 1365 DEGs, respectively, with the majority (~70%) downregulated, suggesting an initial inhibitory effect on gene expression. Functional enrichment analysis indicated that PE-MP exposure primarily affected developmental programs, particularly suppressing those associated with the central and peripheral nervous systems, including brain development, sensory organ development, neural transmission, and synapse function, thereby demonstrating neurotoxic effects. The upregulated processes were predominantly associated with translation, ribosomal function, and cellular stress responses. Notably, 14-day exposure resulted in only 110–190 DEGs, with divergent responses between concentrations, suggesting the potential recovery or adaptation of zebrafish larvae [80].

Cai et al. (2025) demonstrated that early exposure to PS-MPs induces ADHD-like behaviors (hyperlocomotion and impulsive bursts) in zebrafish larvae. Both 0.1 μm and 5 μm particles triggered behavioral effects with stronger transcriptomic dysregulation observed with smaller particles. Mechanistically, pathway analysis revealed that PS-MPs dysregulate GPCR signaling and dopamine-related genes. Since dopamine signaling operates through GPCRs, this suggests that PS-MPs disrupt dopaminergic neuron development as a primary driver of behavioral toxicity. Interestingly, shared dysregulations across particle sizes involved receptor signaling and cytokinesis pathways, indicating both common and size-dependent molecular mechanisms underlying PS-MPs neurotoxicity [47].

Pedersen et al. observed that PS-NPs at 200 nm produced a non-behavioral dose (100 ppb) and a behaviorally effective dose (1000 ppb), resulting in hyperactivity in zebrafish larvae. Transcriptomic profiling revealed 734 DEGs at 100 ppb and 864 DEGs at 1000 ppb with approximately 60% upregulated across both doses and ~50% overlap between conditions. Pathway enrichment analysis of 200 nm NP exposures identified neurological disease, endocrine system disorders, and organismal injury as the most significantly affected disease categories. Nervous system sub-pathways included movement disorders, neuromuscular disease, neuronal morphology, and neurogenesis, implicating key genes such as *eef*2*k*, *gmfb*, *ntf*3, and *csnk*2*b* in synaptic and neurodevelopmental dysfunction alongside *myhb* and *chrna1* in neuromuscular impairment. Beyond neurological effects, both concentrations dysregulated metabolic (*pdk*2*a*, *apoa*4*b.*3, *insrb*), cardiovascular (*myh*6, *amot*, *slc*8*a*1*b*), hepatic (*cyp*3*a*65, *cyp*7*a1*), and gastrointestinal (*aqp*1*a.*1, *mfge*8*b*) pathways, indicating systemic and multi-organ molecular disruption. Notably, alterations in epigenetic regulators (*kdm*2*aa*, *hdac*3, *smyd*3) suggested that NP exposure may also influence gene expression through chromatin remodeling mechanisms with potential long-term transcriptional consequences [81].

Yang et al. (2025) demonstrated that long-term (40 days) PS-MP (1 μm, 25 μg/L) exposure induced neurotoxicity and led to depression-like behavior in adult zebrafish. The 1 μm particles can cross the blood–brain barrier in zebrafish and accumulate in brain tissue. RNA-seq analysis revealed 466 DEGs with enrichment in circadian rhythm and rhythmic processes pathways and in inflammation (*il-*6*, il-*1*β*). Interestingly, among the 466 identified DEGs, 70 overlapped with human depression-related genes. Possibly, a disruption of circadian rhythm can mediate the development of depression-like behavior after MP exposure [82].

One mechanism underlying the neurotoxicity of MNPs is endocrine disruption, in which MNPs interfere with hormone signaling pathways that regulate brain development, neurotransmission, stress responses, and neuronal survival [83,84]. PS-MPs have been shown to induce nitric oxide (NO)-mediated neurotoxicity by disrupting neurotransmitter levels and suppressing *bdnf* gene expression, thereby triggering downstream neuronal apoptosis through the modulation of apoptosis-regulatory molecules in zebrafish embryos [84]. Molecular docking demonstrated that styrene exhibits affinity for Bdnf, p53 and Bcl-2 proteins [84].

Furthermore, PS-MPs of size 10 µm induced seizure behavior in zebrafish larvae. The expression of seizure- and gamma-aminobutyric acid (*GABA*)-related genes increased after PS-MP exposure [85]. GABA is the main inhibitory neurotransmitter in the brain [86]. Intriguingly, only 10 µm PS-MPs produced a seizurogenic effect, whereas smaller sizes accumulated in the brain to a greater extent [85].

Smaller PS-NPs have been shown to accumulate to a greater extent in the brain than larger particles. Exposure to 50 nm and 200 nm PS-NPs revealed distinct gene expression patterns in the zebrafish brain despite inducing a similar number of DEGs. Shared enriched pathways included oxidative stress, inflammatory response, and cytokine–cytokine receptor interaction. Notably, 50 nm PS-NPs showed a greater enrichment of immune-response pathways, along with pathways associated with the visual system, suggesting more severe effects in the zebrafish brain than 200 nm particles [87].

Saputra et al. (2025) found that PS-NPs act as endocrine disruptors, causing behavioral alterations in larval zebrafish. PS-NP exposure resulted in the downregulation of *th*1, *th*2, *ddc*, and *dat*, reducing dopamine synthesis and transport and leading to a loss of TH-positive neurons. Apoptosis-related genes *tp*53*, casp*3*, casp*9*,* and *bax* were upregulated. PS-NPs may interfere with estrogen signaling, which is important for neurodevelopment [88].

Lucon-Xiccato et al. (2026) investigated the effects of biodegradable polybutylene adipate terephthalate (PBAT) and PE-MPs on the learning abilities and cognitive flexibility of adult zebrafish. After 20 days of dietary administration of both MP types, they found a decrease in *c-fos* expression; notably, PE-MPs induced a more pronounced downregulation of *c-fos* than PBAT [89]. *C-fos* can serve as a genetic marker of neural activity; for example, *c-fos* has been found to be activated in the zebrafish brain after introducing fish to a new tank [90]. Although MPs affected brain function at a molecular level in zebrafish, these molecular changes did not translate into detectable alterations in cognitive abilities or learning, as measured by the specific tasks [89].

**Figure 3:**
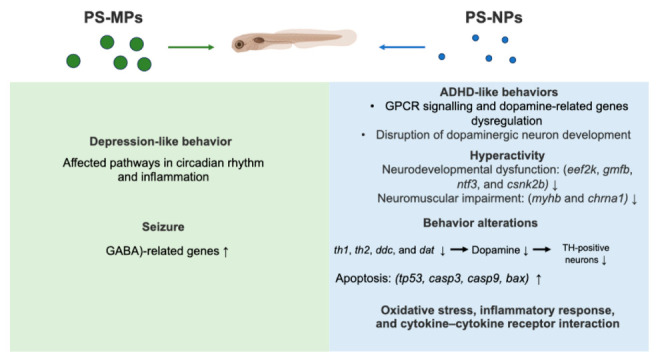
Toxic effects of PS-MNPs on larval zebrafish nervous system.

In the included studies, mostly the effects of PS-type MNPs have been addressed; the altered genes and pathways are summarized in Figure 3.

### 3.4. Visual and Auditory System

The chronic exposure for 45 days to PS-NP of sizes 80, 200 and 500 nm at the environmentally relevant concentration of 0.1 mg/L resulted in hyperactivity and social behavioral deficits in adult zebrafish. Moreover, parental exposure to PS-NP affected the neurobehavior of offspring, resulting in increased mirror attacks, shoaling, and light sensitivity in larvae. Interestingly, the most pronounced effect was caused by 500 nm PS-NPs. Under chronic exposure to 500 nm PS-NPs, PS particles accumulated in the retinal pigment epithelium and photoreceptor cells in adult zebrafish. Additionally, the same changes were observed in offspring. An RNA-seq analysis of eye tissue revealed a total of 1259 genes dysregulated in zebrafish treated with 500 nm PS-NP. Enrichment analysis highlighted pathways related to eye development, such as visual perception, lens development in camera-type eyes, and the structure constituent of the eye lens. KEGG enrichment indicated an altered phototransduction, adipocytokine signaling pathway, cytokine–cytokine receptor interaction, gap junction, TGF-β signaling pathway, and melanogenesis [34]. The TGF-β signaling pathway appeared the most enriched and plays crucial roles in developmental processes in the eye [91]. TGFβ can promote lens fibrosis via the regulation of matrix metalloproteinase expression [92]. Mechanistically, PS-NPs might induce oxidative stress and inflammatory cascades, which, together with the upregulation of cytokines, may drive photoreceptor cell apoptosis and retinal pigment epithelium degeneration, whereas dysregulated TGFβ signaling could promote lens fibrosis via ECM deposition [34]. Probably, alterations in zebrafish neurobehavior could be partially caused by the worsening of vision. MNPs can interact with the eye surface, causing ocular surface inflammation and damage, inducing apoptosis, and reducing corneal and conjunctival epithelial cell viability [93].

Additionally, Saputra et al. (2025) demonstrated that PS-NPs downregulated key visual development genes (e.g., *six*6, *pax*2, *pax*6*a*, and *pax*6*b)* and induced visual dysfunction in zebrafish larvae. Notably, co-treatment with vitamin E had a protective effect on the visual system [94].

Surgical face masks are made of PP and tend to release nano- and micro-fibers (NMFs). Masseroni et al. (2025) studied the effect of NMFs derived from the artificial photodegradation of surgical face masks on zebrafish embryos. The authors observed morphological impairments, including a reduction in eye area. Short-term (6 days) exposure to NMFs resulted in 65 DEGs; predominantly, the downregulated genes involved in energy-related metabolic processes, including acetyl-CoA biosynthesis, pyruvate metabolism, and the tricarboxylic acid cycle (TCA), while the upregulating genes were involved in the negative regulation of developmental processes, such as *bcl*11*aa* and *vsx*1, which are essential for neurodevelopment and eye function. Medium-term exposure (14 days) identified 325 DEGs, with the downregulation of genes involved in sulfotransferase activity and guanosine triphosphate binding, which is a critical cofactor for protein synthesis and mitochondrial physiology, alongside the upregulation of pyruvate and propanoate metabolism pathways, which are indicative of a compensatory energy stress response. The downregulation of *pdha*1*a*, which is crucial for converting pyruvate to acetyl-CoA before TCA cycle entry, directly correlated with impaired mitochondrial respiration and ATP production, potentially explaining the reduced eye development and behavioral alterations through insufficient energy availability for developmental and neurological processes [95].

Sung et al. (2026) explored the effect of PS-NPs on auditory systems. scRNA-seq of zebrafish embryos showed that exposure to 20 nm of PS-NPs for 2 days at 10 µg/mL revealed an increase in hair cells along with enlarged otoliths. Hair cells facilitate otolith formation by providing structural surfaces and secreting mineralization factors. Otoliths are inner ear biominerals that detect gravity, acceleration, and sound. Due to the limited number of hair cells, analysis was focused on otic vesicle cells. This yielded 21 distinct cellular clusters, including otic vesicle cells. DEG analysis of the otic vesicle cell cluster revealed eight upregulated genes: *col*1*a*1*a*, *tmsb*1, *krt*4, *fgfbp*2*b*, *s*100*a*10*b*, *cytl*1, *krt*5, *fstl*1*a*, and *rpl*38. Enrichment analysis revealed pathways such as keratinization and intermediate filament organization. These findings suggest that stress-responsive repair mechanisms may promote hair cell development and otolith enlargement [96]. In contrast, prior studies in zebrafish embryos exposed to higher NP concentrations (25 and 50 mg/L) of comparable size (25 nm) reported decreased otolith size and reduced hair cell numbers [97]. This highlights that the effects of NPs on inner ear development are dose-dependent. Possibly, at higher concentrations, more pronounced inflammation and oxidative stress suppress compensatory stress response mechanisms.

**Figure 4:**
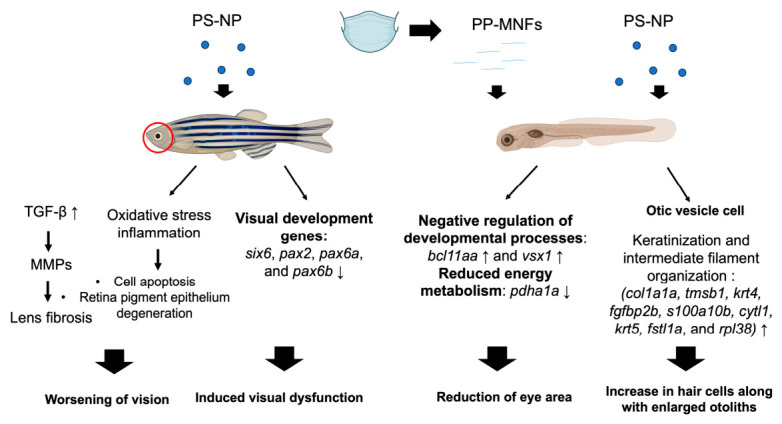
Toxic effects of PS-NPs and PP-MNFs on the visual and auditory systems of adult and larvle zebrafish.

The studies presented indicate that MNPs can affect the normal functions of sensory systems, such as the visual and auditory systems; the mechanisms by which MNPs influence these systems are summarized in Figure 4.

### 3.5. Intestine

Yuan et al. investigated the effects of 1, 10, 100, and 1000 μg/L PE-MPs exposure on adult zebrafish for 7 days and revealed slight damage to intestinal mucosal cells. Using qRT-PCR, they observed an increased expression of genes such as *tnfsf*13, *il*4, *pigr*, *cd*80, *il*10, *ccr*9*b*, *cd*28, *cxcl*12*a*, and *ighv*4-5, which was related to the intestinal immune network of mucosal cells [98]. Jin et al. (2018) demonstrated a size-dependent effect, in which nanoscale polystyrene microplastics (0.5 μm) elicited significantly a greater mRNA upregulation of pro-inflammatory genes (*IL*1*α*, *IL*1*β*, *Ifn*) in the zebrafish gut compared to microscale particles (50 μm) [38].

To assess the size-dependent effects of PS-MPs, Gu et al. (2020) conducted an scRNA-seq study of specific intestinal cell populations in zebrafish. Adult zebrafish were exposed to 100 nm, 5 μm, and 200 μm PS-MPs (500 μg/L) for 21 days. Phagocytes showed the greatest number of DEGs, followed by lymphocytes and enterocytes, while secretory cells were uniquely sensitive to 100 nm MPs. The 100 nm PS-MPs had the most pronounced biological effects—particularly on enterocytes (enriching detoxification and antioxidant pathways) and secretory cells (altering molecular transport and chemotaxis). In contrast, 5 and 200 μm particles primarily triggered immune response alterations with 200 μm particles showing heightened effects on lymphocytes. Additionally, PS-MP treatment remodeled immune populations by increasing T-cell abundance while reducing M1 macrophage proportions. M1 macrophages displayed the most extensive transcriptional disruption (160 DEGs) with nanoscale particles specifically disrupting reactive oxygen species metabolism and NOD-like receptor signaling. In immune cells, all three PS-MP sizes commonly disrupted phagosome function, immune regulation, and cell chemotaxis through the downregulation of key genes (*Cybb*, *Ctss*2.1, *Itgb*2). However, size-specific mechanisms emerged: 100 nm particles altered oxygen transport and hemoglobin expression; 5 μm particles disrupted lysosomal function; and 200 μm particles affected cell surface receptor signaling. Overall, together with an observed increase in pathogenic bacterial abundance, the data point to altered mucosal defense and immune competence as primary consequences of PS-MP exposure. The authors suggest that the broader changes in cells caused by 100 nm might be due to the ability of nanoscale MPs to enter the systemic circulatory system more easily than microscale MPs [56].

Xue et al. (2021) examined the time-dependent effects of PE-MPs in adult zebrafish by analyzing the intestinal transcriptome after 1, 5, and 10 days of exposure to PE-MPs (45–53 μm, 0.6 mg/L). KEGG pathway enrichment of DEGs indicated that carbohydrate and lipid metabolism were the predominant pathways affected by MPs in intestinal tissue [99]. In the intestine, after 1 day, the response was dominated by acute stress and immune activation, marked by the upregulation of *gadd*45*ba* (p53-linked DNA damage/stress signaling) and *socs3a* (immune regulatory signaling), which was consistent with an early defensive reaction to environmental challenge. By 5 days, increased *fbp*2 expression suggested a metabolic adjustment toward greater energy mobilization to sustain the stress response [99]. In contrast, after 10 days, the transcriptome shifted toward a broad suppression of metabolic functions: most DEGs were downregulated and clustered in amino acid and lipid/sphingolipid pathways, demonstrating patterns consistent with starvation-like effects. Interestingly, DEGs were not shared across time points, supporting a staged progression from early stress signaling to longer-term metabolic disruption, with immune involvement persisting and intensifying at 10 days [99].

Pak et al. (2025) examined the combined effects of a lipid-rich diet and PS-NP exposure across zebrafish development from larvae to adults. PS-NPs accumulated mainly in the proximal and distal intestine rather than systemically with active ingestion causing more localized intestinal deposition than passive early-life uptake. However, passive uptake during early development caused greater developmental delay and mortality. A transcriptomic analysis of juvenile zebrafish exposed to environmentally relevant PS-NP concentrations (4.4 ppb) revealed a differential expression of genes mainly related to lipid metabolism, immune and inflammatory responses, oxidative stress, cell proliferation, and embryonic development [40]. To resolve cell-type-specific transcriptomic alterations, the researchers reanalyzed scRNA-seq data from the intestinal tissues of adult zebrafish exposed to 100 nm and 200 nm PS-NPs [56]. They identified reductions in “proliferating cell” and “immune-responding cell-2” in PS-NP-treated groups. PS-NP accumulation resulted in the dysregulation of immune response genes and led to an increased number of phagocytic cells in the adult zebrafish intestine. Another novel finding was that a lipid-rich diet protected the zebrafish intestine from PS-NP accumulation. Feeding a lipid-rich diet markedly reduced intestinal PS-NP accumulation and rescued a subset of PS-NP-induced transcriptomic changes, particularly genes in glycolysis (*adra*2*b*, *gas*8, and *pfn*2*a*), lipid metabolism/membrane remodeling (*hdlbpa*, *slc*1*a*2*b*, and *hspa*4*l*), oxidative stress regulation (*oxr1b*, *dkk3b*, and *gstk1*), and anti-inflammatory responses (*fl*3, *klf*8, and *tent*5*ab*). A possible mechanism is that PS-NPs trigger immune activation, which in turn disrupts lipid metabolism, and that dietary lipids provide compensatory metabolic buffering to mitigate these effects [40].

**Figure 5:**
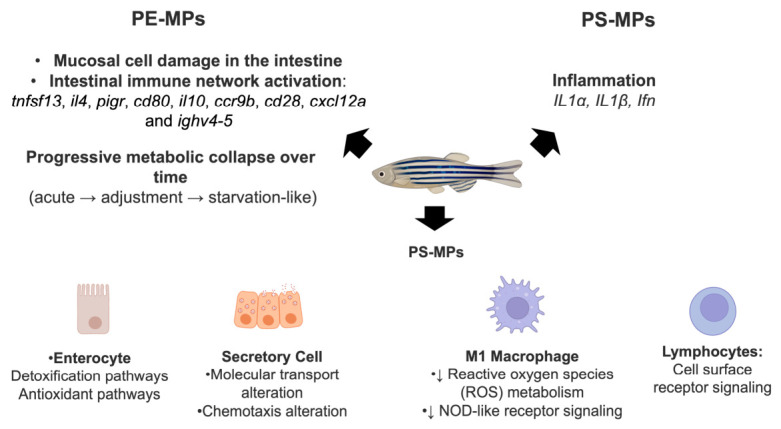
Toxic effects of PE- and PS-MPs exposure on zebrafish intestine.

The genes, pathways and cell types altered by PE- and PS-MP exposure are summarized in Figure 5.

### 3.6. Gills

The aforementioned study by Xue et al. (2021) examined the gill transcriptome under the same conditions: after 1, 5, and 10 days of exposure to PE-MPs (45–53 μm, 0.6 mg/L). In the gills, the main affected pathways were immune response and lipid metabolism. At 5 days, complement-related genes such as *c*3*a*, *c*9, and *cfhl*4 were upregulated, indicating immune activation. In contrast, several chemokine-related genes, including *cxcl*19, *cxcl*8*a*, *cxcl*8*b.*1, *and cxcr*4*a*, were downregulated, suggesting the suppression of chemokine signaling. These opposing responses suggest that PE-MP exposure activated complement-mediated immunity while inhibiting chemokine-driven inflammatory signaling in gill tissue [99].

In general, acute exposure to PE-MPs does not significantly threaten fish life. However, in the long term, reduced metabolism and immune changes, probably attributable to physical damage to the fish gills and intestine, might suppress fish growth and reproduction and increase susceptibility to infection. Xue et al. (2022) found that PE-MPs have size-dependent effects on transcriptomic changes in zebrafish gills. The researchers exposed zebrafish to three MP size ranges (45–53 μm, 90–106 μm, and 250–300 μm) for 5 days and conducted a comparative transcriptomic analysis coupled with physiological assessments. They identified 60, 344, and 802 DEGs following exposure to small, medium, and large MPs, respectively, indicating that larger MPs induced more pronounced molecular alterations in gill tissue. Functional enrichment analysis demonstrated that these DEGs were significantly associated with FoxO signaling, cellular senescence, circadian rhythm regulation, and p53 signaling. Additionally, exposure to medium- and large-sized MPs resulted in cell cycle inhibition and the suppression of apoptosis. At the physiological level, all MP treatments induced oxidative stress, evidenced by significantly elevated glutathione content, while acetylcholinesterase and Na^+^/K^+^-ATPase activities were significantly reduced across all size treatments, suggesting impaired neurotransmission and ion regulation. These molecular and physiological perturbations collectively indicate that MP exposure compromises essential gill functions, including osmoregulation, ion homeostasis, and respiration, with larger particles eliciting more severe effects [37].

Thus, taken together, exposure time and MP size are important factors affecting the toxicity of MP on the gill transcriptome.

### 3.7. Liver

The liver serves as a primary hub for xenobiotic biotransformation, detoxification, storage, and immune responses. MP-induced inflammation increases reactive oxygen species (ROS) production, thereby exacerbating oxidative stress in hepatic cells and leading to impaired hepatic function [100]. Moreover, the liver is connected to the intestine via the portal vein, representing two-way crosstalk among the intestine, its resident microbiota, and the liver itself [101]. The integrity of this axis relies on three key components: a balanced gut microbial community, an intact intestinal barrier, and proper hepatic immune function [101].

Chen et al. (2025) investigated the impact of PS-NP exposure on zebrafish larvae livers. They found a dose-dependent decrease in *miR-122* expression. *miR-122* targets *P53*, whose downstream target is *TIGAR*. The expression levels of *p53* and *TIGAR* were enhanced. P53 and its downstream effector TIGAR play a key regulatory role in the glycolytic and pentose phosphate pathways. PS-NPs caused a gradual decrease in pyruvic acid and lactate levels, while glucose and NADPH levels increased. Thus, PS-NPs induced the downregulation of *miR-122* and shifted glucose metabolism from the EMP to the PPP. The EMP is the primary pathway for rapid ATP generation, and its inhibition significantly reduces energy supply, lowers ATP, and limits the precursors needed for cell growth and repair. Overactivation of the PPP can overproduce NADPH, reducing oxidative stress but causing metabolic imbalance [102].

Adult male zebrafish fed 5 μm PS-MPs at 20 and 100 μg/L via diet for 21 days exhibited dose-dependent reductions in body weight and condition factor, alongside impaired hepatic energy metabolism, with reduced glucose and α-ketoglutaric acid levels. Transcriptomic analysis identified 1388 differentially expressed genes, which were predominantly involved in carbon, lipid, and amino acid metabolism. Key genes involved in glucose and lipid metabolism were downregulated, and GO analysis indicated a disruption of small-molecule catabolism, carboxylic acid metabolism, oxidoreductase activity, and endoplasmic reticulum functions. Overall, PS-MP exposure impaired hepatic energy production and lipid homeostasis, likely contributing to reduced growth [53].

Tian et al. (2024) studied the effect of PP-MP (5 μm) exposure on adult zebrafish. After 14 days of exposure to PP-MPs, intestinal sections showed thinning of the intestinal wall, loss of goblet cells and cilia, and mucosal defects (more severe at 600 mg/L than at 300 mg/L of PP-MPs), while the liver displayed cellular damage and inflammatory infiltration. Additionally, PP-MP exposure resulted in microbiome dysbiosis. Hepatic RNA-seq revealed a widespread downregulation of genes (499 and 707 DEGs) at 300 mg/L and 600 mg/L, respectively, with the common suppression of pathways for cell cycle, DNA replication, homologous recombination, Fanconi anemia pathway and DNA repair [36]. Upregulated pathways were related to metabolic processes such as the TCA cycle, fatty acid degradation and pentose and glucuronate interconversions. Interestingly, correlation analyses linked specific gut taxa to these downregulated hepatic pathways: a positive association between Enterobacteriaceae and the cell cycle, DNA replication, homologous recombination, DNA repair, and the Fanconi anemia pathway was identified. These results suggest a disruptive gut–liver axis interplay with pentose and glucuronate interconversions [36].

TWP exposure caused size- (100–120 and 250–380 μm) and time-dependent (15 and 90 days) disruption of the zebrafish liver transcriptome. Short-term exposure induced stronger transcriptional responses than long-term exposure, with larger TWPs producing more DEGs than smaller TWPs, suggesting an acute stress response that partially stabilized over time, possibly indicating an adaptive response. Larger TWPs mainly affected peptide and amide metabolism and activated immune pathways such as NOD-like receptor, C-type lectin receptor, and MAPK signaling, indicating inflammation and innate immune activation. Smaller TWPs more strongly disturbed organic acid and energy-related metabolism with a pronounced enrichment of immune and antiviral pathways, which are potentially linked to microbiota dysbiosis. After long-term exposure, both particle sizes showed broader metabolic reprogramming and cellular adaptation, although larger TWPs continued to disrupt protein metabolism, while smaller TWPs mainly affected protein transport and cellular homeostasis [103]. Similar results were reported by Zhang et al. (2025), who found that TWP exposure induced size- and time-dependent transcriptomic alterations in zebrafish liver with larger particles consistently producing greater numbers of DEGs and more enriched GO terms across both exposure periods (15 and 30 days). Key disrupted pathways included xenobiotic biodegradation, lipid metabolism, carbohydrate metabolism, and cofactor/vitamin metabolism with a notable downregulation of fatty acid degradation genes and PPAR signaling [104].

Limonta et al. investigated the effects of virgin HD-PE and PS-MPs on adult zebrafish fed at environmentally relevant concentrations of 100 and 1000 μg/L for 20 days. Liver transcriptomic analysis identified 326 differentially expressed genes, indicating largely overlapping yet dose-dependent responses. The main effects included the dysregulation of immune pathways, downregulation of genes involved in antimicrobial defense and epithelial integrity, and suppression of energy metabolism, particularly steroid and terpenoid biosynthesis. These molecular changes were consistent with histopathological alterations, including epithelial detachment, mucus hypersecretion, and increased neutrophil infiltration in the gut and gills. MP exposure also altered circadian behavior, suggesting broader physiological disruption [105].

Chronic exposure (80 days) to thermally aged, bottle-derived PET MPs resulted in liver damage and steatosis in adult zebrafish. Transcriptomic profiling of the liver after 7 days of exposure to thermally aged PET (h-PET) and normal PET (n-PET) at 100 μg/L revealed that h-PET had a more pronounced effect on the transcriptome. Pathways upregulated in h-PET were uniquely identified and related to glucose metabolism (e.g., PI3K-Akt signaling and insulin resistance) and to fatty liver disease and inflammation (TLR/NF-κB pathway). The authors suggested that h-PET7 damages the intestinal barrier, causing dysbiosis and LPS translocation, which trigger systemic inflammation and activate hepatic de novo lipogenesis through the PI3K/Akt and SREBP-1c pathways, leading to hepatic steatosis via insulin resistance [101]. This study demonstrates how storage conditions can amplify the harmful effects of MPs.

ScRNA-seq revealed that 28-day PS-NP exposure (100 nm, 500 ng/mL) induced hepatocyte dysfunction through sex-specific and shared responses. Male hepatocytes showed a greater disruption of lipid metabolism, whereas female hepatocytes were more sensitive to estrogen and mitochondrial stress. Both sexes exhibited a dysregulation of PPAR signaling. Immune cells showed distinct responses: lymphocytes upregulated inflammatory pathways (*nfkbiaa*, *irf*7, *IL*1*β*, *IL*6, *TNFα*, *IFNα*, T cell activation) and Herpes simplex virus 1 infection pathways, whereas macrophages downregulated ferroptosis, apoptosis, and pattern recognition signaling. Both immune cell types showed reduced lysosomal/phagosomal function, impairing PS-NP degradation. Non-parenchymal cells (HSCs, cholangiocytes, endothelial cells, epithelial cells, erythrocytes) exhibited altered gene expression in oxidative phosphorylation, autophagy, and metabolic pathways [55].

Farmed fish are commonly supplemented with a high-fat diet (HFD) as a strategy to enhance energy intake and partially substitute dietary protein requirements [106,107]. However, it has been found that the HFD causes a bioaccumulation of contaminants [108].

Du et al. investigated HFD administration together with PS-MP at an environmentally relevant concentration (1000 μg/L) over a 14-day exposure in juvenile zebrafish. The 5 μm PS-MPs accumulated more in the liver of HFD zebrafish than in controls, whereas the 50 μm PS-MPs remained gut-localized at similar levels in both groups. Both 5 and 50 μm MP exposure increased hepatic lipid accumulation. Exposure to 5 μm MPs caused the most severe accumulation in the HFD group. RNA-seq showed altered pathways related to xenobiotic biodegradation and metabolism, carbohydrate metabolism, amino acid metabolism, lipid metabolism, and PPAR [109]. The same research group found that smaller PS-NPs (0.05 to 0.1 μm) had a similar effect on juvenile zebrafish administered an HFD. Interestingly, intestinal tissue was affected only in HFD zebrafish with PS-NP treatment, showing intestinal epithelial cell damage and a decrease in the number of goblet cells [110]. RNA-seq showed similar altered pathways in the liver; however, in this case, oxidative phosphorylation appeared to be the most enriched pathway. Additionally, whereas 5 and 50 μm MPs showed enrichment in the fatty acid degradation pathway, 0.05 to 0.1 μm PS-NPs led to fatty acid biosynthesis [110]. Overall, these two studies suggest that PS-NPs aggravate the harmful effects of HFD. MPs may modulate hepatic lipid metabolism in zebrafish through the regulation of lipid-associated signaling pathways. Interestingly, 40 DEGs overlapped between the childhood obesity-related DEGs and PS-MP-associated genes identified in the liver of juvenile zebrafish [111]. This supports the implementation of zebrafish in transcriptomic studies of MNPs’ toxic effects.

Hua et al. (2024) exposed zebrafish embryos to environmentally relevant concentrations of PP-MPs (0.4 and 50 mg/L) from 2 hpf to 120 hpf. Zebrafish showed reduced spontaneous movement. The transcriptomic profiling of zebrafish larvae exposed to PP-MPs revealed pronounced, dose-dependent gene expression changes: RNA-seq detected 786 and 1105 DEGs in the 0.4 mg/L and 50 mg/L groups, respectively, with 248 DEGs shared between treatments. Specifically, at 0.4 mg/L, the most significantly affected biological processes were ADP metabolic processes, glycolytic processes, and pyruvate metabolic processes, whereas at 50 mg/L, cellular components such as ribosomes, mitochondrial respirasomes, proton transporting ATP synthase complexes, and respiratory chain complexes were substantially enriched. At the mitochondrial level, genes encoding electron transport chain complexes I through IV showed concentration-dependent downregulation, which was accompanied by compromised mitochondrial ultrastructure characterized by broken cristae, shallower matrices, and ruptured membranes. These disruptions in mitochondrial energy metabolism may be associated with impaired behavior in zebrafish larvae. Additionally, a disturbance of glycolysis/gluconeogenesis pathways was confirmed by increased glucose content and might be associated with body weight gain in zebrafish larvae [112].

The study by Zhao et al. (2021) used smaller-sized PE-MPs (1–4 μm) and environmentally relevant concentrations (10, 100, and 1000 μg/L) on the early life stages of zebrafish. The authors observed changes in glucose metabolism with increased glucose content in the larvae’s bodies and decreased pyruvic acid. The total cholesterol and triglyceride content increased only in the group of larval zebrafish exposed to 1000 μg/L of PE-MPs, and the total bile acid content was elevated accordingly. qRT-PCR analysis showed a reduced expression of glucose metabolism-related genes: *pepckc* decreased in both the 100 and 1000 μg/L groups, whereas *PK*, *HK*1, and *GK* decreased only in the 1000 μg/L-treated group. Levels of cholesterol and triglycerides are closely related to liver function, and imbalances in these substances can lead to disorders of lipid metabolism in the liver [113]. Levels of genes involved in triglyceride synthesis, such as *ACC*1, *FAS*, *SREBP*1, *CPT*1, and *FABP*6, were significantly decreased in the 1000 μg/L-treated group. Any disbalance in triglycerides might be enhanced by dysregulated gut microbiota caused by PE-MP exposure. Additionally, changes in the mRNA levels of these phospholipid-related genes, such as *acp6*, *selenoi*, *xkr*7, *slc*27*a*1*a*, and *ptdss*1*a*, corroborated the changes in phospholipids observed in the metabolomic analysis. Overall, 7-day exposure to PE-MPs at low, environmentally relevant concentrations disturbs important metabolic pathways, such as glucose and lipid metabolism, indicating that PE-MPs could affect the embryonic development of zebrafish [114].

**Figure 6:**
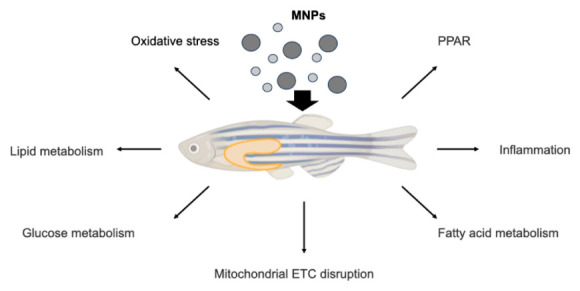
Processes in the zebrafish liver affected by MNPs exposure.

The processes affected by MNPs exposure are summarized in Figure 6.

### 3.8. Reproductive System

PS-NPs have been shown to cause neurobehavioral and reproductive toxicity with sex-specific differences in zebrafish [115]. PS-MPs enhanced the expression of pro-fibrogenic genes such as *tgfβ*1, *ctgf*, *fibronectin, col*1*a*1*a* and *col*1*a*1*b* in the ovary tissue of zebrafish

PS-MP exposure caused gonadal morphological alterations in female zebrafish. PS-MP treatment led to alterations in genes related to brain steroidogenesis, ovary steroidogenesis, the *sirt*1–*p*53 pathway, cellular stress response, and vitellogenesis. Molecular docking revealed that PS-MPs exhibit binding affinity for SIRT-1 and estrogen receptors (ERα and ERβ). The binding of MPs to these receptors can cause competitive inhibition and their dysfunction [116].

Zheng et al. (2024) observed that PS-NPs caused a marked, dose-dependent impairment of reproductive function in zebrafish. Egg production and fertilization rates were significantly reduced across all treatment groups with the most pronounced effects at 500 nm compared with 80 nm and 200 nm. A transcriptomic analysis of ovarian tissue from zebrafish exposed to 500 nm at an environmentally relevant concentration (0.5 mg/L) for 30 days revealed 729 dysregulated genes. Pathway enrichment analyses consistently highlighted a significant disruption of lipid-related biological processes, with notable alterations in the PPAR signaling pathway and multiple lipid metabolism pathways, including alpha-linolenic acid, linoleic acid, ether lipid, and arachidonic acid metabolism. Additionally, complementary non-targeted metabolomic profiling identified 226 significantly altered metabolites, of which 35% were implicated in lipid metabolism [117]. As nuclear receptors, PPARs are key regulators of lipid metabolic processes, inflammatory responses, and cellular differentiation [118]. Based on complementary omics analyses, it can be suggested that PS-NPs caused ovulatory dysfunction by disrupting PPAR and lipid metabolism [117].

Existing studies confirm that maternal exposure to PS-NPs can lead to their transfer to fish offspring [119,120].

Cao et al. (2025) investigated the molecular mechanisms by which maternal exposure to PS-NPs (50 nm) disrupts embryonic development. First, it was found that 120 days of exposure to PS-NPs at environmentally relevant concentrations (10 and 100 μg/L) resulted in morphological abnormalities and behavioral changes in zebrafish offspring, including reduced head area, body length, and locomotor activity. RNA-seq analysis of offspring at 0 hpf identified 3468 shared DEGs between the maternal 10 and 100 μg/L europium-chelated PS-MP (PS-Eu) exposure groups, while 886 and 2107 DEGs were uniquely identified in the maternal 10 μg/L and 100 μg/L PS-Eu exposure groups, respectively. Among enriched pathways, oxidative phosphorylation was the most suppressed pathway in both maternal PS-NP exposure groups. Mitochondria play a crucial role in energy production. Thus, the inactivation of mitochondrial pathways resulted in lower oxygen consumption rates, which likely contribute to the phenotypic defects caused by maternal exposure [120].

In a study conducted by Llewellyn et al. (2025), PS-NPs induced hyperactive swimming behavior in both F0 larvae, directly exposed during early development for 5 days, and their unexposed F1 offspring, suggesting a transgenerational effect. Additionally, F0 adults exhibited reproductive deficits. A transcriptomic analysis of gonadal tissues in males and females revealed that all tested concentrations (100, 1k and 10k ppb) caused alterations in the transcriptome. Overall, male tissues had more altered genes than female tissues. Endocrine system disorder and tissue morphology pathways were uniquely and predominantly enriched in male gonads with a non-monotonic pattern of DEGs across NP concentrations. Endocrine-related sub-pathways were categorized into endocrine cancers, hormone signaling, endocrine tissue physiology, and other endocrine diseases such as diabetes mellitus and infertility [121].

Sendra et al. (2022) assessed the effects of MPs derived from surgical face masks at different stages of degradation. Different stages of face mask degradation produced distinct transcriptomic effects in zebrafish larvae even though the masks came from the same original material. Exposure to poorly degraded products (PDM) altered gene expression, with enrichment in isoprenoid/sterol biosynthesis and redox processes, whereas highly degraded products (HDM) caused broad immune suppression. Water derived from HDM (W-HDM) triggered a surprising overexpression of hemoglobin/heme-binding genes, suggesting altered oxygen transport or a hypoxic response [122]. All MP types altered the expression of genes related to reproductive processes with HDM showing the greatest effect [122].

### 3.9. Effect on Regeneration

Fish and other multicellular organisms are delicate, making tissue and organ damage an unavoidable part of their lifespan. For this reason, the ability to restore tissue architecture and function is indispensable, and any breakdown in this process could jeopardize their survival and reproductive success in the wild. Organisms that have been injured are thought to be more vulnerable to the effects of environmental contaminants. Thus, it is worth studying the effects of MP during the regenerative process.

Gu et al. (2020) investigated caudal fin regeneration in zebrafish larvae during PS-MP exposure. PS-MPs were detected around the amputation zone, implying particle engagement with tissues bordering the injury. Fin amputation induced the expression of most regeneration-related genes. For RNA-seq analysis, zebrafish were treated with 1 mg/L of PS-MPs of different sizes (50 and 500 nm) for 12 and 72 h post-fertilization (hpa). RNA-seq analysis revealed a total of 1066 DEGs at 12 hpa, with fewer at 72 hpa, correlating with morphological recovery timelines. The 50 nm particles induced more DEGs than the 500 nm particles at 12 hpa (534 vs. 121 DEGs), but this pattern reversed at 72 hpa, indicating that particle size influences the timing and magnitude of transcriptional disruption. Interestingly, PS-MP exposure predominantly downregulated genes, contrasting sharply with amputation-induced upregulation, demonstrating suppressive effects on regenerative transcription. Genes in critical regeneration pathways (Wnt, FGF, IGF, BMP, Hh, RA, Notch) were upregulated post-amputation but downregulated following microplastic exposure particularly with 50 nm particles. Moreover, substantial overlap existed between MP-induced DEGs and amputation-responsive genes, confirming that MPs specifically target regeneration-essential molecular machinery. KEGG analysis highlighted the disruption of biosynthesis/metabolism pathways across treatments with a microplastic-specific enrichment of PPAR signaling, ECM–receptor interactions, retinol metabolism, and peroxisome pathways, suggesting metabolic reprogramming and matrix remodeling interference as key toxicity mechanisms [32]. These results show that PS-MP exposure inhibited the regenerative capacity of zebrafish larvae that underwent amputation.

### 3.10. Effect on Immune System

Veneman et al. (2017) studied the effect of PS-MP injected into zebrafish embryos during early developmental stages. Exposure was conducted by internal yolk injection in zebrafish: at the blastula stage and at 2 dpf, which was followed by observation up to 5 days post-injection (dpi). After blastula injection, most particles remained at the injection site, whereas injection at 2 dpf resulted in greater vascular redistribution. A transcriptomic analysis revealed distinct temporal gene expression patterns, with 26 particle-specific differentially expressed genes at 1 dpi and 51 genes at 3 dpi, demonstrating systemic immune responses despite limited particle distribution. The primary adverse effect observed was activation of the innate immune system, specifically through upregulation of the complement cascade via the alternative pathway, as evidenced by an increased expression of genes including *cfb*, *cfh*, *c*3*a.*2, *c*3*a*.3, *c*3*a.*6, and *c*9. Additional pathways significantly enriched included nuclear receptors in lipid metabolism and toxicity at both time points and oxidative stress at 3 dpi. The mechanistic connection between transcriptomic changes and adverse effects was demonstrated through confocal imaging, which confirmed the uptake of PS-MPs by neutrophils and macrophages, directly linking complement system activation with immune cell recruitment and phagocytic responses. These molecular initiating events suggest that PS particles trigger immunological recognition processes that could lead to uncontrolled complement activation, potentially resulting in downstream tissue damage and inflammatory responses [39].

MNPs exposure can disturb immune homeostasis by inducing oxidative stress and altering inflammatory gene expression, which can suppress immune defense and increase vulnerability to bacterial infection [123,124].

Yang et al. (2024) studied the effects of PS-NPs (100 nm) and PS-MPs (5 μm) on zebrafish embryos and larvae. NPs uniquely reduced the embryo hatching rate, elevated the embryo heart rate, and caused an expansion of the endoplasmic reticulum in kidney tissue. MPs induced extensive lipid droplet accumulation in kidney tissue and caused greater increases in both ROS levels and triglyceride content than NPs. NPs uniquely dysregulated five pathways, including the cytosolic DNA-sensing pathway, two signal transduction-related pathways, and arachidonic acid metabolism, with a total of 126 DEGs. MPs activated the Toll-like receptor signaling pathway among three significantly altered pathways with a comparatively lower total of 104 DEGs. Both NPs and MPs significantly elevated embryo and larval mortality, compromised antibacterial resistance against *E. piscicida*, suppressed antioxidant enzyme activity, and increased oxidative stress markers. Both NPs and MPs commonly disrupted the C-type lectin receptor signaling pathway and arachidonic acid metabolism pathway, with predominantly downregulated gene expression and shared downregulation of the key immune factor *il1b*, collectively indicating convergent immunosuppressive and lipid metabolic dysfunction. These findings suggest that particle size influences MNP toxicity, as larger particles are associated with lipid accumulation, while smaller ones more frequently induce ER stress [124]. C-type lectin receptor signaling, Toll-like receptors, and cytosolic DNA-sensing pathways are all part of innate immunity [125,126,127]. The disruption of these pathways might be associated with increased vulnerability to bacterial infection [124].

As the most abundant circulating leukocytes, neutrophils are key effectors of innate immunity, rapidly mobilizing and transmigrating through the vascular endothelium to sites of infection or injury upon inflammatory stimulation [128].

Wang et al. (2025) demonstrated via RNA-seq that a 96 h exposure of zebrafish larvae to 100 μg/L of 0.1 μm MPs enriched pathways associated with ROS generation, neutrophil migration, and histone lactylation. Mechanistically, MP exposure elevated H3K18 lactylation, an epigenetic modification driven by lactate, which is the terminal product of glycolysis. Lactylation, in turn, induced ROS production via DUOX and promoted neutrophil recruitment. Moreover, oxidative stress was found to reciprocally regulate H3K18 lactylation, establishing a positive feedback loop that further amplifies neutrophil mobilization [129].

Mechanistic studies often rely on commercially produced MPs because their physicochemical properties (size, shape, and surface characteristics) can be tightly controlled, allowing the contribution of each parameter to be tested. However, these model particles typically have uniform sizes, defined surface chemistries, and predominantly spherical shapes, which can differ substantially from environmentally derived microplastics in both physical and chemical behavior, limiting ecological realism and direct extrapolation to real-world exposures. Thus, more environmentally relevant MPs need to be tested to provide more realistic results in in vivo behavioral and toxicological experiments. Bacteria can colonize MP surfaces to form biofilms. Missawi et al. (2024) studied the involvement of bacteria in shaping realistic MPs hazards in zebrafish embryos. The authors assessed the fate and toxicological behavior of MPs associated with *Aeromonas salmonicida achromogenes* in zebrafish embryos exposed to PET micro-fragments and micro-fibers. Zebrafish embryos were exposed to PET-MPs at a concentration of 1000 μg/L after 2 hpf for 3 days. Among the negative effects of PET-MPs is the induction of earlier embryo hatching. Exposure to PET-MPs combined with bacteria significantly delayed the hatching rate compared to the groups exposed to single MPs. The authors suggested that bacteria may mask the effects of MPs when adsorbed to their surface. To analyze the effect of PET-MPs and the presence of bacteria, they checked, with qRT-PCR, the expression of genes related to the immune system (*mpx*), inflammation (*nfkb*2, *tfa*), oxidative stress (*sod1*, *cat*, *cyp*1*a*), metabolism (*apoeb*, *fasn*, *cs*) and apoptosis (*bcl*2) in zebrafish larvae. The levels of *cyp*1*a* and *sod* were significantly decreased in the larvae of all exposure groups. *Apoeb*, *mpx*, *nfkb*2 and *tfa* expressions were significantly downregulated in the larvae of bacteria-exposed groups, and no significant changes in the expression of the above indicators were observed when combined with fibers or fragments and in MP-exposure groups. Thus, no synergistic effect was observed when MPs were combined with *A. salmonicida*. Bacterial exposure alone caused immune suppression and an inhibition of inflammatory response, while PET-MPs caused a disruption of the oxidative stress pathway only [52]. Zheng et al. found that untreated MPs caused greater dose- and time-dependent intestinal damage and inflammation than MPs degraded by microorganisms. This observation was seen at the transcriptomic level: untreated MPs caused the highest expression of *il-*1*β*, *il*-8 and *TNF-α*. Therefore, the toxicity of MPs can be potentially reduced after microbial degradation [130].

## 4. Discussion

The toxicological outcomes of MNPs exposure are shaped by their distinct physico-chemical properties, including polymer type, particle size, and exposure concentration, leading to diverse transcriptomic responses in aquatic organisms such as zebrafish.

The transcriptomic studies that applied bulk- and sc-RNA studies and were discussed in this review are summarized in Table 1.

### 4.1. MNP Type

Both PE-MPs and PS-MPs are ubiquitous in the environment and have been extensively studied for their potential toxic effects across various organisms and cell types [133,134]. Here, we want to discuss the toxicity effect of different MNP types in zebrafish and its transcriptome.

MNPs have diverse chemical structures, and these differences are important when interpreting their biological effects. For example, in vitro experiments showed that PE particles (approximately 1 μm) are transported more efficiently through a simulated intestinal barrier than PS particles [135]. To date, the literature we found focuses on the toxicity of distinct MNP types, sizes, and concentrations rather than on comparing the toxic effects of different MNP types under the same exposure conditions. However, comparing MNP effects in similar settings could provide insights into the biological effects of MNPs.

The study by Liu et al. (2026) compared the effects of exposure to PS-MPs and PE-MPs in adult zebrafish. PS-MPs produced a more pronounced reduction in body weight than exposure to PE-MPs. Both plastic types reduced heart rate in a similar manner with reduced myocardial cell density, cardiac tissue lesions, and cell apoptosis. Moreover, PS-MPs induced a greater reduction in heart rate and more severe impairment of myocardial cell density. Both PS-MPs and PE-MPs altered the expression of cardiac-related genes, resulting in the upregulation of *tnnt*2*a*, *appa*, and *caspase-*3 and the downregulation of *nkx*2.5, *myh*6*,* and *atp*2*a*2 [136]. For example, the reduced expression of *nkx*2.5, a crucial transcription factor for cardiac development, can indicate impairment of the structural and functional integrity of the heart [137]. The downregulation of *myh*6, which encodes the heavy chain of cardiac myosin, leads to decreased myocardial contractility [138]. The upregulation of *caspase-*3, a protease that plays a key role in apoptosis, indicates the initiation of the apoptotic program by cells [139]. Possibly, PS-MPs may exhibit increased cardiac toxicity compared to PE, potentially due to the presence of the benzyl (phenyl) ring in their chemical structure, which contributes to different interactions with cells and/or the biomolecules inside cells.

Another study comparing TWP and LAP toxicity in zebrafish larvae found that under identical conditions, there were both decreased heart rate and increased malformation rates with LAP leachate showing a stronger inhibitory effect on neurodevelopment. RNA-seq revealed that LAP induced about 3000 DEGs, whereas TWP altered around 200 DEGs with 93 genes overlapping. KEGG enrichment analysis revealed that TWP mainly enriched metabolic pathways, with ferroptosis and necroptosis indicating cellular stress, while LAP leachate enriched mostly metabolic pathways, plus signaling, translation, and endocrine-related pathways. Treatment with ferrostatin-1, a ferroptosis inhibitor, ameliorated MP-induced phenotypes in both groups, identifying ferroptosis as a key mechanism underlying MP toxicity [131].

Meng et al. (2025) compared heavy-duty vehicle TWPs (HTWPs) with light-duty vehicle TWPs (LTWPs) in zebrafish. HTWPs exhibited rougher surfaces and sharper edges than LTWPs. HTWP exposure induced more pronounced oxidative stress than LTWP exposure. HTWP exposure resulted in 4305 DEGs compared with 1818 DEGs under LTWP exposure. HTWPs predominantly disrupted carbohydrate homeostasis, whereas LTWPs triggered compensatory lipid metabolic responses. Both TWP types converged on inflammatory pathway dysregulation, including MAPK signaling and C-type lectin receptor pathways. These differences may be attributable to the higher concentrations of bioavailable metals, such as Zn, Fe, and Al, in HTWP leachates compared with LTWP leachates [132].

Based on the reported studies, PE-MPs at environmentally relevant concentrations (10–1000 μg/L) and at high concentrations (5 and 20 mg/L) caused no severe effects on the development of embryos and larvae. Adverse effects included gut microbiome dysbiosis and alterations in lipid and carbohydrate metabolism [80,114]. In contrast, exposure to 1000 μg/L of PET-MPs caused impairments such as an increased early hatching rate, smaller body length, and a reduced eye area in the early life stages of zebrafish, whereas for PE-MPs at the same concentration, no developmental abnormalities were reported. PS-MPs at 100 μg/mL caused behavioral alterations associated with ADHD [47], and those at 10 mg/L caused severe morphological alterations such as tail alterations, pericardial oedema, and yolk sac deformities [140]. In adult stages, PE-MPs also showed lower toxicity. For example, at low concentrations, with a maximum of 1000 μg/L, PE-MP exposure caused damage to mucosal cells and gut microbiome dysbiosis [98], and in another study, an increased respiratory rate was reported [99]. PS-MP exposure also caused gut microbiome dysbiosis, increased mucin secretion and gut inflammation [38,56], reduced body weight and condition factor, and disrupted hepatic energy metabolism [53]. Although the toxicity of PS-MNPs on zebrafish has been reported as more pronounced, in studies with approximately similar exposure conditions, PE resulted in a higher number of DEGs [47,80].

PP-MPs also resulted in larval developmental impairments: for example, reduced eye area [95], and at concentrations of 0.08 and 10 mg/L, it induced an increased heart rate, increased body weight, and behavioral alterations [112].

PP and PE share relatively similar chemical structures. However, PP appears to exert more pronounced effects during both early and adult zebrafish life stages compared with PE [36,112]. Possibly, the methyl side group in PP’s molecular backbone may alter its surface properties and interactions with biological membranes. Notably, under similar exposure conditions in larval zebrafish, PE-MP exposure resulted in a higher number of DEGs compared with PP exposure. This might be attributed to particle size: the PP-MP particles were two to seven times smaller than the PE particles [80,112]. Additionally, differences in RNA-seq data quality and data processing can influence the number of identified DEGs.

In adult zebrafish, hepatic and intestinal samples have been investigated more frequently than other tissues. We identified studies evaluating the effects of PP-, HDPE-, and PS-MPs on the liver of adult zebrafish. In particular, PP- and PS-MPs were comparable in size with exposure durations of 14 and 21 days, respectively. The PP-MP exposure concentration was approximately 3- to 6-fold higher than that used for PS-MPs [36,53]. Nevertheless, PP-MP exposure induced fewer DEGs—approximately 3-fold fewer than PS-MP exposure. This suggests that the number of transcriptomic alterations may not be determined by exposure concentration alone but also by polymer type, particle surface properties, uptake efficiency, additive composition, or bioavailability. Moreover, the affected biological pathways differed between polymers: PP-MPs were mainly associated with DNA damage and cell cycle-related pathways, whereas PS-MPs and co-exposure to HDPE and PS predominantly affected metabolic processes such as steroid and fatty acid metabolism. These findings indicate that different polymer types may induce distinct molecular responses in adult zebrafish liver [36,53].

Overall, the main limitation to comparing the effects of different MNP types is the different exposure settings of zebrafish to MNPs, such as time, concentration and particle size.

### 4.2. Effect of Exposure Time and MNP Size on Zebrafish Transcriptome

Some studies used RNA-seq to investigate gene expression across different exposure times. Interestingly, longer exposure periods yielded fewer DEGs. For example, after 12 days of exposure, ~1500 DEGs were identified in a transcriptomic analysis of the whole larval body, whereas after 14 days, only ~100–200 DEGs were identified. The authors suggest this may be due to partial recovery and adaptation to PE-MPs [80]. In contrast, in larvae that underwent MP injection, 26 DEGs were obtained after 1 dpi and 51 DEGs were obtained after 3 dpi. This may reflect the number of DEGs in the mock-injected group, as after 3 days, the larvae had recovered from the tissue damage caused by injection. While at 1 dpi, pathway enrichment analysis identified processes related to responses to various chemical and biological compounds; at 3 dpi, pathways were enriched for immune response [39].

In the intestine of adult zebrafish, the number of identified DEGs increased consistently with days of exposure. This pattern can be explained by MPs accumulating in the intestine over time. In the gill transcriptome, around 400–500 DEGs were identified on the first and tenth days of exposure, whereas on the fifth day, fewer than 100 DEGs were observed. Pathway enrichment analysis suggests a possible mechanism involving activation of the immune response on the first and fifth days, which was followed by the adaptation and reconstruction of metabolism induced by the organism on the tenth day [99].

Gu et al. (2020) conducted a study with two exposures and two PS-MP sizes in a larval model with an amputated caudal fin. For smaller particles (50 nm), more DEGs were identified at 12 hpa than at 72 hpa. By contrast, the opposite effect was observed for 500 nm particles [32]. This demonstrated that transcriptomic alterations also depend on the size of MNPs: larger particles can irritate tissue to a greater extent than smaller ones, persist in tissue for longer, and cause more severe inflammation and ROS production, which aligns with the expression of genes involved in oxidative stress and cytokine genes [32].

Among the physicochemical properties of MNPs, particle size has been widely recognized as the most influential parameter governing their absorption, bioaccumulation, and biodistribution, and it consequently plays a determining role in mediating their toxic effects in biological systems [141].

A size-dependent pattern of MNP tissue distribution has been reported in adult zebrafish, with smaller particles capable of penetrating deeper tissues, including the gut and liver, whereas larger particles remain confined to the gill and digestive tract [142,143]. In the study by Ding et al. (2020), larger MP particles have been associated with more pronounced oxidative stress responses, an effect attributed to their greater capacity to cause mechanical tissue injury, which subsequently triggers downstream disruptions in biochemical signaling pathways [144].

A clear size-dependent effect has been observed in the gills of adult zebrafish: the number of identified DEGs was positively correlated with the PS-MPs size [37]. Notably, pathways differed across PE-MP sizes. Shared pathways in the 250–300 μm and 90–106 μm treated groups included cellular senescence, p53 and FoxO signaling, which can regulate the cell cycle in response to stress [37]. In the group treated with smaller particles, different pathways were enriched [37].

In the scRNA of intestinal tissue from adult zebrafish, 100 nm PS-NPs induced the broadest molecular response compared with 5 and 200 nm particles. Notably, secretory cells are disproportionately responsive to NPs, whereas phagocytes mount a largely size-independent response, which is consistent with their specialized role in particle clearance. Lymphocytes show a broader response to 200 µm particles, possibly reflecting mechanical stress-induced immune signaling, whereas enterocytes display a more balanced, distributed response across all sizes [56]. Greater effects on the transcriptome can be explained by the higher accumulation of 5 and 100 nm particles in intestinal tissue compared with 200 nm particles [56].

In larval zebrafish, MPs and NPs exposure resulted in similar numbers of DEGs [124]. The affected pathways in both groups were related to pathogen recognition, and specifically for NP, the FoxO and MAPK signaling pathways were enriched, which might point to activation of the innate immune response [124]. The lack of observable differences in DEG profiles may be explained by the comparatively low exposure concentration employed (1 mg/L), which may not have been sufficient to drive differential transcriptional regulation between treatment groups.

Interestingly, PS-NPs and PS-MPs differentially perturb neurodevelopmental gene networks with NPs predominantly disrupting myelinogenesis and cholinergic signaling, while MPs more strongly affect neurotrophic factor expression and axonal guidance [79]. PS-NPs drove the transcriptional upregulation of antioxidant genes (e.g., *nfe*2*l*2*a*, *sod*1, and *cat*), reflecting an active compensatory oxidative stress response, whereas PS-MPs had a less pronounced effect on transcriptional changes [79].

### 4.3. Overall Effect of MNPs on Zebrafish Transcriptome

MNPs exert a significant and complex influence on gene expression, leading to widespread transcriptomic alterations that reflect cellular stress, metabolic disruption, and developmental impairments [145]. Although some studies do not distinguish between upregulated and downregulated genes, a prevalent trend indicates an inhibitory effect on gene expression across various biological processes. MNPs exposure frequently results in the downregulation of genes critical for fundamental organismal development, particularly in larval zebrafish. This includes genes associated with brain, head, and eye development with a notable impact on the central nervous system. Such downregulation suggests a disruption of normal developmental trajectories and processes. However, the response is not exclusively inhibitory; there can be a selective upregulation of certain genes, such as those involved in heart development, in response to PS-NP exposure, even alongside the downregulation of others [136]. This indicates a nuanced and sometimes opposing regulation of gene expression, highlighting the context-dependent nature of MNP-induced effects.

Beyond developmental processes, transcriptomic analyses reveal that MNPs can trigger a broad suppression of metabolic functions with many differentially expressed genes downregulated and clustering in amino acid and lipid/sphingolipid pathways, mimicking starvation-like effects [53,99]. This suggests that MNPs interfere with essential metabolic machinery. Furthermore, MNPs induce cellular stress programs and disrupt signaling pathways and regulatory networks, including those involved in oxidative stress, inflammatory responses, endocrine disruption, neurotoxicity, DNA damage, apoptosis, and overall metabolic homeostasis [57,81,105]. The specific impact on gene expression can vary over time, progressing from early stress signaling to longer-term metabolic disruption.

Additionally, transcriptomic studies of MNP effects in zebrafish may have translational relevance. For example, zebrafish models can provide insight into obesity, as PS-MP exposure altered 40 genes that also overlap with transcriptomic data associated with childhood obesity [111].

### 4.4. Mechanisms of MNPs Toxicity

The primary mechanism by which MNPs cause toxicity is oxidative stress. Several interconnected mechanisms may underpin MNP-induced oxidative stress. One key mechanism is the impairment of mitochondrial electron transport chain function, leading to electron leakage and the overproduction of ROS [146]. In parallel, PS-NPs trigger membrane phospholipid peroxidation, which not only undermines membrane structural integrity but also serves as a source of secondary ROS, further perpetuating oxidative damage [147]. Moreover, MNPs can adsorb and bind biomolecules, thereby dysregulating intracellular signaling cascades, attenuating antioxidant defense mechanisms, and interfering with redox-sensitive transcription factors, including NF-κB and Nrf2 [111,148].

One of the most commonly enriched pathways across several studies was PPAR. PPAR has mostly been found to be dysregulated in liver tissue but also in caudal fin and ovarian tissue (Table 1). PPAR stands for peroxisome proliferator-activated receptors, which are nuclear receptors regulating glucose metabolism, lipid metabolism, and inflammatory responses [149]. The PPAR family consists of three subtypes: PPARα, PPARβ/δ, and PPARγ. PPARs orchestrate the transcriptional regulation of a broad array of genes that govern key hepatic processes, including metabolic and energy–homeostatic pathways, oxidative stress responses, inflammatory signaling, and apoptotic mechanisms [150]. In the liver, the activation of PPARα enhances fatty acid oxidation and thermogenic processes. PPARα is a nutrient-sensing receptor and can be increased during food restriction [151]. PPARα can promote the expression of certain transcription factors (e.g., *FIAF* and *FGF21*), thereby increasing circulating free fatty acids and ketone bodies to supply energy [152,153]. PPARα was overexpressed under PS-MPs and a high-fat diet [109], whereas in another study, *PPARα* was found to be downregulated under only PS-MPs exposure [53]. However, conditions of exposure were relatively similar except those regarding the high-fat diet. Possibly, the overexpression of PPARα under a high-fat diet and PS-MPs can be regarded as a compensatory response to excess lipid load. Excessive lipid ligands activate *PPARα, promoting* β-oxidation [154]. Under PS-MP exposure alone, zebrafish may experience hepatic stress, impaired nutrient metabolism, oxidative damage, inflammation, or mitochondrial dysfunction. These effects can suppress normal lipid-catabolic signaling, leading to the downregulation of PPARα and reduced fatty acid oxidation [53]. Additionally, it can also lead to the downregulation of PPARα and PPARγ, which can participate in suppressing inflammation by inhibiting NF-κB signaling which results in decreased inflammatory cytokine production [155]. Moreover, PPAR-γ modulates the differentiation and maturation of adipocytes [156]. The downregulation of PPAR-γ can result in lower fat synthesis and accumulation that can lead to reduced body weight [53].

**Figure 7:**
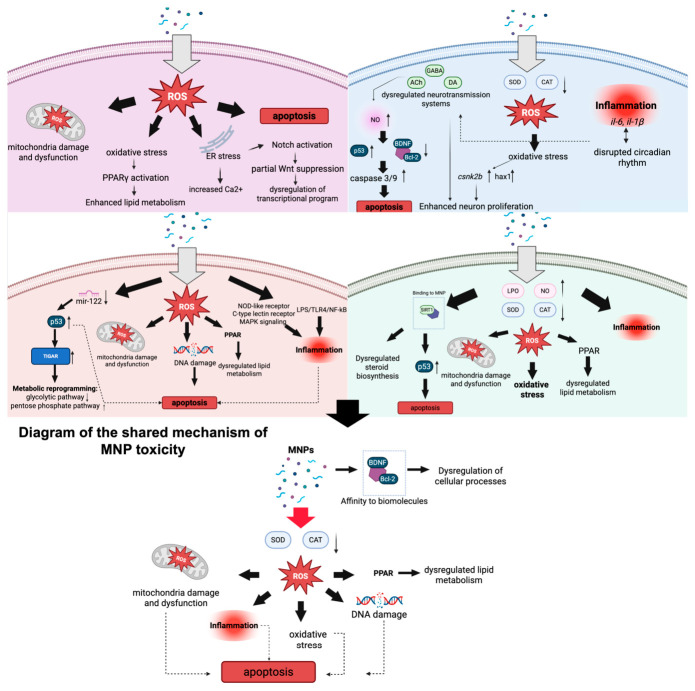
Proposed organ-specific and shared mechanisms of micro- and nanoplastic (MNP)-induced toxicity. Based on the reviewed studies, MNPs exposure may induce toxic effects in the heart, brain, liver, and reproductive system through several interconnected cellular mechanisms. Across these organs, one of the most commonly reported responses is the excessive generation of reactive oxygen species (ROS), accompanied by reduced antioxidant defense capacity, including altered superoxide dismutase (SOD) and catalase (CAT) activity. Increased ROS production may lead to oxidative stress, mitochondrial damage and dysfunction, DNA damage, inflammation, the dysregulation of lipid metabolism, and the activation of apoptotic pathways. In organ-specific contexts, MNPs exposure has also been associated with altered calcium signaling and endoplasmic reticulum stress in the heart; the disruption of neurotransmission and circadian rhythms in the brain; metabolic reprogramming and changes in lipid-related signaling pathways in the liver; and impaired steroidogenesis and reproductive dysfunction. Overall, the diagram summarizes both organ-specific effects and shared mechanisms, suggesting that oxidative stress, mitochondrial impairment, inflammation, DNA damage, metabolic dysregulation, and apoptosis are central pathways involved in MNP-induced toxicity.

Here, based on the included studies on MNP toxicity in the heart, brain, liver, and reproductive system, we attempted to create a general diagram of the cellular mechanisms that mediate the harmful effects of MNPs (Figure 7). The diagram integrates organ-specific findings into a unified framework, highlighting shared pathological pathways, including oxidative stress, inflammatory cascades, mitochondrial dysfunction, and apoptosis, that collectively mediate the harmful effects of MNPs exposure across biological systems. It should be noted that not all included studies explicitly described the cellular or molecular mechanisms underlying MNP-induced toxicity, which limited the comprehensiveness of the proposed diagram. Therefore, further well-designed studies are warranted to fully elucidate the specific signaling pathways, molecular targets, and downstream effectors that are disrupted upon MNPs exposure.

Computational docking revealed that tea bag-derived MPs exhibit binding affinity for proteins encoded by genes essential for normal development—*Zhe1*, *Sod1*, and *p53*— all of which were found to be transcriptionally altered in zebrafish larvae after MP exposure [157].

Overall, the effect of MNPs on gene expression is multifaceted, and it is marked by the widespread downregulation of developmental and metabolic genes alongside the targeted upregulation of others, reflecting the cell’s intricate attempt to respond to and cope with xenobiotic exposure. These transcriptomic shifts are critical for understanding the underlying mechanisms of MNPs toxicity and the resulting phenotypic changes observed in affected organisms.

### 4.5. Limitations

A limitation of this review is the variability in the settings and methodologies of the RNA-seq experiments included. This variation includes differences in experimental design, sequencing depth, sample size, data-processing pipelines, normalization methods, and DEG screening criteria, including the selected fold-change and significance thresholds. Therefore, comparisons of DEG profiles and pathway enrichment results across studies should be interpreted with caution. Because the number and identity of DEGs directly influence downstream functional enrichment analyses, such variability may result in differences in the enriched signaling pathways reported across studies. In addition, the use of different annotation and pathway databases, such as GO, KEGG, and Reactome, may further contribute to inconsistencies in pathway interpretation. Therefore, comparisons of DEG profiles and pathway enrichment results across studies should be interpreted with caution. Future studies using standardized sequencing protocols, bioinformatic workflows, enrichment databases, and DEG selection thresholds would improve comparability and strengthen the interpretation of molecular mechanisms associated with MNPs exposure.

## 5. Conclusions

This review indicates that MNP toxicity in zebrafish is strongly influenced by polymer type, particle size, exposure duration, concentration, and tissue-specific accumulation. Transcriptomic evidence shows that MNPs exposure disrupts key biological processes, including development, lipid and energy metabolism, immune and inflammatory responses, oxidative stress regulation, and apoptosis. Among the recurrent molecular mechanisms, oxidative stress and the dysregulation of metabolic pathways, particularly PPAR signaling, appear to play central roles in MNP-induced toxicity. However, differences in experimental design across studies limit direct comparisons, highlighting the need for standardized exposure models and integrated multi-omics approaches to better define the mechanisms of MNP toxicity.

## Figures and Tables

**Table 1:** Transcriptomic studies of MNPs exposure in larval and adult zebrafish.

Method Sample	Conc.	MNP	Time	Enriched Pathways	DEGs	Ref.
Early life stages of zebrafish						
RNA-seq of whole body	5 mg/L and 8 mg/L	NP 20 nm	2 days	5 mg/L: nuclear cell cycle DNA replication, epithelial–mesenchymal cell signaling, positive regulation of neutrophil activation; polyamine transmembrane transport, negative regulation of neutrophil activation and negative regulation of mitochondrial membrane permeability; 8 mg/L: centriole elongation, nucleotide excision repair DNA gap filling, and negative regulation of glycogen metabolism; regulation of polyamine transmembrane transport, hyaloid vascular plexus regression	N/A	[60]
scRNA-seq tail				HSPCs: ribosome biogenesis, rRNA processing, hydrogen peroxide metabolism, antioxidant activity and peroxidase activity; ECs: mitotic cell cycle progression, organelle fission, mitotic nuclear division and antioxidant/redox-regulatory activities tubulin binding, antioxidant activity, oxidoreductase activity	N/A	
scRNA-seq of whole body	10 µg/mL	PS: 20 nm	3 days	RBC cluster: translation-related pathways, translation-related biological processes, differentiation and heme synthesis	N/A	[49]
RNA-seq of whole body	5 mg/L	PE: 10–45 μm	2 days	DOWN: embryo/organ development; central/peripheral nervous system development. UP: translation/protein synthesis; RNA processing	451 ↑ and 1283 ↓	[80]
RNA-seq of whole body	0.08 and 10 mg/L	PP: 6.30 ± 0.14 μm	5 days	Ribosomes, oxidative phosphorylation, retinol metabolism and glycolysis/gluconeogenesis	0.08 mg/L: 786; 10 mg/L: 1105	[112]
RNA-seq of whole body	0.2 mg/L	PP: 265 nm ± 83 nm	6 days	DOWN: spliceosome, lipoic acid metabolism, TCA cycle, pyruvate metabolism, 2-oxocarboxylic acid metabolism, DNA replication, nucleotide excision repair, basal transcription factors; UP: alanine, aspartate, and glutamate metabolism	52 ↑ and 13 ↓	[95]
RNA-seq of whole body	1 nL containing 5 mg/mL	PS: 2–10 μm	1 and 3 days	Activation of the complement system by alternative pathway	1 day: 26; 3 days: 51	[39]
RNA-seq of whole body	10 μg/mL	PS: 42 nm	5 days	Response to stimulus, developmental process, metabolic process and the immune system, endocytosis, MAPK signaling, calcium signaling pathway, FoxO signaling pathway, TGF-beta signaling, amino sugar metabolism, nucleotide sugar metabolism and purine metabolism	356 DEGs: 140 ↑ and 216 ↓	[57]
RNA-seq of caudal fin	1 mg/L	PS: 50 and 500 nm	12 and 72 h	12 hpa: PPAR signaling pathway, ECM–receptor interaction, retinol metabolism and peroxisome; 72 hpa: PPAR signaling, ECM–receptor and Hh signaling	12 hpa/50 nm: 59 ↑ and 454 ↓; 12 hpa/500 nm: 19 ↑ and 102 ↓; 72 hpa/50 nm: 24 ↑ and 116 ↓; 72 hpa/500 nm: 83 ↑and 170 ↓	[32]
RNA-seq of whole body	100 μg/mL	PS: 0.1 μm and 5 μm	5 days	5 μm: GPCR signaling and neurotransmitter-related pathways, cytochrome-c, cytokine signaling, and peroxidase activity, mitochondria- and ribosome-related pathways; 0.1 μm: reproduction-associated pathways, DNA modification processes, immune-related functions)	5 μm: 979 ↑ and 1547 ↓; 0.1 μm: 864 ↑ and 707 ↓	[47]
RNA-seq of whole body	100, and 1000 ppb	PS: 200 nm	5 days	Organismal injury and abnormalities, endocrine system disorder, neurological disease, skeletal/muscular and nervous system development	100 ppb: 734 ↑ and 500 ↓; 1000 ppb: 864 ↑ and 530 ↓	[81]
RNA-seq of whole body	1 mg/L	PS: 100 nm 5 μm	7 days	100 nm NPs: C-type lectin receptor signaling; phototransduction, FoxO signaling, MAPK signaling, cytosolic DNA sensing, 5 µm MPs: C-type lectin receptor signaling, phototransduction, Toll-like receptor signaling, cytosolic DNA-sensing, caffeine metabolism, arachidonic acid metabolism	100 nm: 52 ↑ and 74 ↓; 5 μm: 50 ↑ and 54 ↓	[124]
RNA-seq of liver	1000 μg/L	PS: 5 and 50 μm	14 days	Ribosome; PPAR signaling; peroxisome; metabolism of xenobiotics by cytochrome P450; pyruvate metabolism; glycolysis/gluconeogenesis; tryptophan metabolism; fatty acid degradation; primary bile acid biosynthesis; beta-alanine metabolism	N/A	[109]
scRNA-seq of whole body	10 μg/mL	PS: 20 nm	10 days	Keratinization and intermediate filament organization	N/A	[96]
RNA-seq of liver	1000 μg/L	PS: 0.05–0.1 μm	7 days	Oxidative phosphorylation; spliceosome; RNA polymerase; fatty acid biosynthesis; retinol metabolism; butanoate metabolism; valine, leucine and isoleucine biosynthesis; riboflavin metabolism; valine, leucine and isoleucine degradation; glycerophospholipid metabolism	N/A	[110]
RNA-seq; scRNA-seq of whole body	4.4 ppb	PS: 100 nm and 200 nm	14 days	Lipid metabolism and catabolism, immune responses and inflammatory pathways, oxidative stress regulation, cellular proliferation, and embryonic development	N/A	
RNA-seq of whole body	TWP and LAP: 150–180 nm	2.5 g/L	5 days	TWP: iron–ion binding, ferroptosis, steroid hormone biosynthesis, metabolism of xenobiotics–cytochrome P450; LAP: teroid hormone biosynthesis, retinol metabolism, metabolism of xenobiotics–cytochrome P450, PPAR signaling	TWP: 126 ↓ and 72 ↑; LAP: 2468 ↓ and 750 ↑	[131]
RNA-seq of whole body	PDM: 7.37 and 12.22 µm; HDM: 1.44 and 954.90 µm	10 mg/L	10 days	PDM: reproduction, immune/inflammatory response, and lipid/sterol metabolism; HDM: reproductive pathways, RNA silencing, heat shock and stress response, immune/inflammasome, metabolic and coagulation pathway; W-HDM: oxygen transport/hypoxia and hemopoiesis, immune/inflammatory response, MAPK signaling, protein processing in the endoplasmic reticulum, RNA degradation, stress/HSP70 response, hemoglobin/heme-binding processes, and cell proliferation/extracellular matrix remodeling	PDM: 58 ↓ and 105 ↑; HDM: 197 ↓ and 90 ↑; W-HDM: 23 ↓ and 80 ↑	[122]
RNA-seq of whole body	PS: 50 nm	10 and 100 μg/L	120 days	10 μg/L: lysosome, pyrimidine metabolism, phagosome, nitrogen metabolism, oocyte meiosis, and RNA polymerase; 100 μg/L: p53 signaling pathway, one carbon pool by folate, base excision repair, non-homologous end-joining, nucleocytoplasmic transport, and homologous recombination; 10 and 100 μg/L: oxidative phosphorylation, Fanconi anemia pathway, cell cycle, nucleotide excision repair, mismatch repair, ubiquitin mediated proteolysis, spliceosome, SNARE interactions in vesicular transport, and DNA replication	10 μg/L: 2714 ↑ and 1640 ↓; 100 μg/L: 3298 ↑ and 2277 ↓	[120]
**Adult life stages**						
RNA-seq of intestine	PE: 46–53 μm	0.6mg/L	1, 5, and 10 days	1 day: p53 signaling pathway, adipocytokine signaling pathway; 5 days: pentose phosphate, fructose and mannose metabolism, glycolysis/gluconeogenesis, cardiac muscle contraction; 10 days: Notch signaling pathway, lysine degradation, MAPK signaling, sphingolipid metabolism, glycerolipid metabolism, mitophagy–animal	1 day: 6 ↓; 5 days: 5 ↓; 10 days: 170 ↑ and 16 ↓	[99]
RNA-seq of gills	PE: 45–53 μm, 90–106 μm, and 250–300 μm	10,000 particles/L	5 days	250–300 μm: amino sugar and nucleotide sugar metabolism, arachidonic acid metabolism, prolactin signaling pathway, adipocytokine signaling pathway, circadian rhythm, p53 signaling pathway, osteoclast differentiation, TNF signaling pathway, C-type lectin receptor signaling pathway, cytokine–cytokine receptor interaction, FoxO signaling pathway, cellular senescence, NOD-like receptor signaling pathway; 90–106 μm: p53 signaling pathway, circadian rhythm, cellular senescence, FoxO signaling pathway; 45–53 μm: phenylalanine metabolism, ubiquinone and other terpenoid-quinone biosynthesis, lysosome, complement and coagulation cascades, antigen processing and presentation	45–53 μm: 11 ↑ and 49 ↓; 90–106 μm: 229↑ and 115 ↓; 250–300 μm: 446 ↓ and 356 ↓	[37]
RNA-seq of liver	PP: 5 μm	300 and 600 mg/L	14 days	Chromosome segregation, mitotic cell cycle processes, DNA repair, and responses to DNA damage, cell cycle regulation, DNA replication, homologous recombination, Fanconi anemia pathway, and base excision repair pathways	300 mg/L: 25 ↑ and 474 ↓; 600 mg/L: 74 ↑ and 703 ↓	[36]
RNA-seq of liver	HD-PE and PS: 25–90 μm	100 and 1000 μg/L	20 days	Lipid metabolism (sterol biosynthetic process, steroid metabolic process and fatty acid metabolic process), steroid biosynthesis pathway, terpenoid backbone biosynthesis	1000: 43 ↑ and 158 ↓; 100: 55 ↑ and 195 ↓	[105]
RNA-seq of liver	PS: 5 μm	100 μg/L	21 days	Carbon metabolism: metabolism of xenobiotics by cytochrome P450, carbon metabolism, pyruvate metabolism, ascorbate and aldarate metabolism, glyoxylate and dicarboxylate metabolism, pentose and glucuronate interconversions; amino acid metabolism; lipid metabolism: steroid biosynthesis, fatty acid metabolism, PPAR signaling, steroid hormone biosynthesis, primary bile acid biosynthesis, biosynthesis of unsaturated fatty acids	632 ↑ and 752 ↓	[53]
scRNA-seq of intestine	PS: 100 nm, 5 μm, and 200 μm	500 μg/L	21 days	All sizes: phagosome processes, regulation of immune system processes, and chemotaxis; 5 μm: lysosome function; 100 μm: NOD-like receptor signaling	100 nm: 1209 5 μm: 837 200 μm: 895	[56]
RNA-seq of ovarian tissue	PS: 500 nm	0.5 mg/L	30 days	Lipid-related biological processes, PPAR signaling	391 ↑ and 338 ↓	[117]
RNA-seq of brain and gonadal tissue	PS: 200 nm	100, 1000 and 10,000 ppb	5 days	Males: endocrine system disorder and tissue morphology pathways; Females: organismal injury and abnormalities and neurological disease pathways	Female: gonad (968/233/273), brain (1047/92/97); Male: gonad (3445/1978/3938, brain (2703/414/2129)	[121]
scRNA-seq of liver	PS: 100 nm	500 ng/mL	28 days	Hepatocytes: PPAR signaling, MAPK, actin cytoskeleton, and FoxO signaling, oxidative phosphorylation and metabolic pathways; Macrophage: activation of amino acid biosynthesis, glycolysis, lipid metabolism; Lymphocytes: HSV1 infection, T cell activation pathways; hepatic stellate cells: included oxidative phosphorylation, autophagy—animal, cardiac muscle contraction2, FoxO signaling, mTOR signaling; epithelial cells: drug metabolism—cytochrome P450, metabolism of xenobiotics by cytochrome P450, ECM–receptor interaction, PPAR signaling, and focal adhesion		[55]
RNA-seq of eye tissue	PS: 500 nm	0.1 mg/L	45 days	Visual perception, lens development in camera-type eye, structure constituent of eye lens, transmembrane transport, positive regulation of peptidyl–tyrosine phosphorylation, cGMP binding, phototransduction, adipocytokine signaling pathway, cytokine–cytokine receptor interaction, gap junction, TGFβ signaling pathway and melanogenesis	500 ↑ and 759 ↓	[34]
RNA-seq of liver	HTWPs and LTWP: 100–150 μm	10 mg/L	15 days	HTWP: glycolysis/gluconeogenesis, amino sugar and nucleotide sugar metabolism, fructose and mannose metabolism, starch and sucrose metabolism, pentose phosphate pathway, and galactose metabolism, TNF and MAPK signaling; LTWP: fatty acid degradation, fatty acid beta-oxidation/oxidation, biosynthesis of unsaturated fatty acids, steroid hormone biosynthesis, and glycerophospholipid metabolism, PPAR, C-type lectin receptor, and MAPK signaling	HTWP: 4305; LTVP: 1818	[132]
RNA-seq of brain	PS: 50 and 200 nm	500 μg/L	7 days	Leukocyte chemotaxis, response to abiotic stimulus, granulocyte migration, response to interleukin-1, immune system response, chemokine signaling and IL-17 signaling pathways, sensory perception, response to light stimulus, cone photoresponse recovery, phototransduction, GABAergic synapse and retinol metabolism pathway	200 nm: 151 ↑ and 289 ↓; 50 nm: 270 ↑ and 191 ↓	[87]
RNA-seq of liver	TWP: 100–120 and 250–380 μm	10 mg/L	15 and 90 days	HL15: amide/peptide metabolism and biosynthesis, NOD-like receptor, C-type lectin receptor, glycerophospholipid metabolism, HSV-1 infection; HS15: organic/oxo/carboxylic acid metabolism, immune-related and HSV-1 infection pathways; HL90: regulation of protein metabolism, organonitrogen compound catabolism, intracellular transport, peptide metabolism, amide biosynthesis; HS90: regulation of protein metabolism, organonitrogen compound catabolism, intracellular transport, amide transport, protein transport	HL15: 1560 ↓, 884 ↑; HS15: 1023 ↓, 746 ↑; HL90: 1746 total; HS90: 1329 total	[103]
RNA-seq of brain	PS: 1 μm	25 μg/L	40 days	Phototransduction, circadian rhythm, rhythmic processes, and response to light stimulus	198 ↓ and 268 ↑	[82]

↓—downregulated DEGs; ↑—upregulated DEGs.

## Data Availability

No new data were created or analyzed in this study. Data sharing is not applicable to this article.
